# Dietary supplementation with RG92 improves laying performance and egg quality in laying hens

**DOI:** 10.1016/j.psj.2026.107972

**Published:** 2026-10-17

**Authors:** Shigenori Nobata, Sadahiro Iwabuchi, Fumi Asao, Kenta Oka, Atsushi Tajima, Shigeru Hamada

**Affiliations:** a SARABiO Onsen Probiotic Lab., Co., Ltd., 1356-8, Oaza-Tsurumi, Beppu, Oita, 874-0842, Japan; b Department of Bioinformatics and Genomics, Graduate School of Medical Sciences, Kanazawa University, 13-1, Takara-machi, Kanazawa, Ishikawa, 920-8640, Japan

**Keywords:** Laying hen, Microalga, Laying performance, Short-chain fatty acids, Peripheral blood mononuclear cells

## Abstract

The productive lifespan of commercial laying hens has been extended through genetic improvement; however, age-associated declines in egg production and egg quality remain major challenges for the poultry industry. This study evaluated the long-term effects of supplementation with the microalga Mucidosphaerium sp. RG92 on laying performance and egg quality in laying hens. In an experimental trial, Boris Brown laying hens were maintained as one control flock (10 hens) and one RG92-supplemented flock (10 hens) from 5 to 119 weeks of age. To assess whether a similar pattern was observed under practical commercial farming conditions, a separate field evaluation was conducted using one control flock (309 hens) and one RG92-supplemented flock (289 hens) from 47 to 79 weeks of age. During the experimental trial, the RG92-supplemented flock showed a less pronounced age-associated decline in hen-day egg production, lower proportions of broken and oversized eggs, and a higher proportion of acceptable eggs during the late laying period. Under commercial farming conditions, eggs sampled from the RG92-supplemented flock showed higher albumen height, Haugh unit, and yolk index. To investigate physiological and molecular responses associated with RG92 supplementation, fecal short-chain fatty acid (SCFA) concentrations and peripheral blood mononuclear cell (PBMC) transcriptomic profiles were evaluated. RG92 supplementation was associated with higher fecal concentrations of acetate, propionate, and butyrate. RNA sequencing of PBMCs revealed transcriptomic differences associated with immune regulation and cellular redox homeostasis. Collectively, these observations suggest that RG92 supplementation may help attenuate age-associated deterioration in laying performance and egg quality during prolonged laying and was accompanied by changes in fecal SCFA profiles and PBMC transcriptomic profiles, supporting further evaluation of RG92 as a functional nutritional strategy for laying hens.

## Introduction

The genetic improvement of laying hens has markedly increased egg production over recent decades, enabling extended laying cycles and delaying flock replacement. However, advancing age is accompanied by deterioration of eggshell quality and other age-related declines in egg quality, resulting in an increased incidence of cracked or broken eggs and a reduction in the proportion of marketable eggs (Rayan et al., 2010; Rodriguez-Navarro, et al., 2002). Therefore, maintaining both high egg production and egg quality throughout the late laying period has become increasingly important for sustainable and profitable egg production. Although induced molting has traditionally been used to restore laying performance, conventional fasting-based methods are associated with animal welfare concerns, prompting the development of alternative nutritional and management strategies.

Nutritional interventions using functional feed ingredients have therefore attracted increasing attention as sustainable strategies to maintain productivity and health in laying hens. Functional feed additives are expected not only to improve production performance but also to enhance intestinal health, antioxidant capacity, immune function, and resistance to environmental stress, thereby contributing to prolonged laying persistency and improved egg quality. Among these ingredients, microalgae have emerged as promising feed additives because they contain abundant proteins, lipids, pigments, vitamins, and diverse bioactive compounds. Numerous studies have demonstrated that dietary microalgae supplementation improves laying performance, egg quality, immune responses, antioxidant status, and the nutritional value of eggs (Madacussengua, et al., 2025; Saadaoui, et al., 2021).

*Mucidosphaerium* sp. strain RG92 is a freshwater unicellular green microalga originally isolated from a hot spring in Beppu, Japan. Based on morphological characteristics and phylogenetic analysis of the 18S rRNA gene, RG92 was classified as a member of the genus *Mucidosphaerium* within the family Chlorellaceae (Bock, et al., 2011). Biochemical analyses have demonstrated that RG92 contains abundant glycolipids, particularly digalactosyldiacylglycerol (DGDG), a chloroplast membrane lipid reported to possess anti-inflammatory and metabolic regulatory activities. Experimental studies further showed that RG92 extracts suppress inflammatory responses and oxidative stress in mammalian cells, while dietary supplementation with RG92 improved glucose metabolism and ameliorated insulin resistance in both animal models and human subjects (Kaseda, et al., 2020; Miyata, et al., 2021). These findings suggest that RG92 represents a promising functional microalgal ingredient with potential applications in animal nutrition. Given the reported beneficial effects of microalgal supplementation on laying performance and egg quality in hens (Madacussengua, et al., 2025; Saadaoui, et al., 2021), together with the anti-inflammatory and metabolic regulatory properties previously demonstrated for RG92, we hypothesized that RG92 supplementation might help maintain productivity and egg quality during prolonged laying. However, to our knowledge, no previous study has evaluated the long-term effects of RG92 supplementation on laying performance or egg quality in laying hens. This lack of information therefore represents an important knowledge gap. Therefore, the aim of the present study was to evaluate the effects of long-term dietary supplementation with RG92 on laying performance, egg quality, fecal short-chain fatty acid concentrations, and PBMC transcriptomic profiles in laying hens.

## Materials and methods

### Preparation of RG92 extract

RG92 extract was prepared according to a previously described method (Miyata, et al., 2021). Briefly, RG92 was cultured in a 5-L flask for 3 weeks and harvested by centrifugation (10,000 × g for 10 min). The resulting pellet was suspended in 50 g of ethanol and disrupted by ultrasonication for 20 min, followed by incubation at 4°C for 24 h. Cell debris was removed by filtration through Whatman No. 2 filter paper (Whatman, Maidstone, UK), and the resulting filtrate was used as the RG92 extract. A single batch of RG92 extract was used throughout both the experimental and commercial farm trials and was stored at −20°C until use.

### Animals and experimental design

#### Experimental trial at the SARABiO Institute

Twenty 5-week-old Boris Brown laying hens were obtained from Kajiwara Hatchery (Oita, Japan) and randomly assigned to either the control group (n = 10) or the RG92 group (n = 10). The 10 hens assigned to each treatment were maintained together as a single floor-reared flock; therefore, one flock-level experimental unit was available for each treatment. Birds were housed in a floor-rearing system at the SARABiO Institute, with ad libitum access to feed (Power Layer, JA Zen-Noh Kumiai Feed Co., Ltd., Tokyo, Japan) and water under natural photoperiod conditions. The RG92 group received drinking water supplemented with RG92 extract throughout the experimental period. Based on previous animal studies and taking drinking-water consumption into consideration, the amount of RG92 extract was set at approximately 1/1,250,000 of the total body weight of the flock. Accordingly, one drop of RG92 extract (approximately 0.02 mL) was diluted in 15 L of drinking water and provided to the RG92 group. The experiment continued until 119 weeks of age. Egg production was recorded daily, and no mortality occurred during the experimental period. All experimental procedures were approved by the Animal Care and Use Committee of the SARABiO Institute (approval obtained in 2022) and were conducted in accordance with the relevant Japanese guidelines for the care and use of experimental animals.

#### Commercial farm field evaluation

To assess whether a similar age-associated pattern in laying performance was observed under practical commercial farming conditions, a field trial was conducted at Ueda Poultry Farm (Kashima, Saga, Japan). A total of 309 control hens and 289 RG92-supplemented hens were included in the trial, which was conducted from 47 to 79 weeks of age. Both groups were housed within the same commercial poultry house, with one rearing section assigned to the control treatment and one rearing section assigned to the RG92 treatment. Thus, one flock-level experimental unit was available for each treatment. Both groups were managed under the same commercial production conditions and received the same basal diet. For practical administration to a large commercial flock, RG92 was provided via the feed rather than through drinking water. The supplementation level was determined on the basis of the total body weight of the flock and the feeding protocol established for practical farm use. The daily amount of RG92 extract corresponded to approximately 1/7,000,000 of the total flock body weight (664.7 kg), equivalent to approximately 0.094 g (0.12 mL) of RG92 extract. Specifically, six drops of RG92 extract (approximately 0.12 mL) were diluted in 3 L of water, and the resulting solution was applied as a top dressing to the feed. Hen-day egg production was recorded throughout the trial. Because RG92 supplementation began partway through week 47 and the trial ended partway through week 79, laying-performance analyses were based on data from 48 to 78 weeks of age. Weekly values were summarized as the mean ± SE. This trial was conducted independently of the experimental study at the SARABiO Institute.

### Evaluation of laying performance in experimental trial

Egg production performance was evaluated in the experimental trial conducted at the SARABiO Institute. Egg production was recorded daily throughout the experimental period. Hen-day egg production (%) was calculated as: *Hen-day egg production (%) = (number of eggs produced / number of hens) × 100.* Broken eggs and oversized (LL; ≥70 g) eggs were recorded throughout the experiment. Beginning at 80 weeks of age, all eggs were weighed individually and classified as MS (52-57 g), M (58-63 g), L (64-69 g), or LL (≥70 g). The proportions of broken eggs, LL eggs, and acceptable eggs (defined as non-broken eggs excluding LL-size eggs) were subsequently calculated. Because individual eggs could not be attributed to individual hens, all longitudinal laying-performance variables were calculated from daily flock-level egg counts.

### Evaluation of internal egg quality

Egg quality was evaluated using eggs randomly collected at 79 weeks of age, at the end of the commercial farm field evaluation. Eggshell color was assessed using a shell color fan (Gen Corporation, Gifu, Japan) according to the manufacturer’s scale using five eggs from each of the control and RG92 groups. In addition, five eggs collected from young hens (26 weeks of age) were evaluated as an age-reference group. Internal egg quality was evaluated using 10 eggs per group. Albumen diameter, albumen height, yolk diameter, and yolk height were measured for each egg. The Haugh unit was calculated from egg weight and albumen height, and the yolk index was calculated as the ratio of yolk height to yolk diameter. Because the eggs were obtained from a commercial production flock, only a limited number could be allocated for experimental quality assessment without interfering with routine egg sales; therefore, no formal a priori sample-size calculation was performed, and the numbers of eggs analyzed were determined by practical availability. Because individual laying hens could not be identified, the sampled eggs were regarded as subsamples of the corresponding treatment group rather than as independent biological replicates of the dietary treatment.

### Fecal short-chain fatty acid analysis

Fresh fecal samples were collected from hens in the SARABiO experimental trial between 74 and 77 weeks of age and processed on the day of collection. Fecal samples were collected approximately 1.5 h after daily poultry-house cleaning (8:00 AM) by randomly collecting three fresh fecal droppings from each treatment group, which were immediately placed on ice. Because the hens were housed under floor-rearing conditions and individual droppings could not be assigned to specific hens, the three fecal specimens collected at each sampling occasion were regarded as subsamples of the corresponding treatment group rather than as independent biological replicates. For sample preparation, an appropriate amount of fecal material was weighed and diluted with sterile water to a final concentration of 0.2 g/mL. Samples were homogenized using a BioMasher II (Nippi, Tokyo, Japan), followed by centrifugation at 3,000 × g for 10 min. The resulting supernatants were used for organic acid analysis. Concentrations of lactate, acetate, propionate, butyrate, total SCFAs, and total organic acids were determined according to the method described (Inoue, et al., 2019). Briefly, samples were derivatized with 2-nitrophenylhydrazine and analyzed by high-performance liquid chromatography using a YMC-Pack ODS-AM column (250 × 4.6 mm; YMC Co., Ltd., Kyoto, Japan). Total SCFA concentration was calculated as the sum of acetate, propionate, and butyrate, and total organic acid concentration was calculated as the sum of the measured organic acids.

### RNA quality assessment, library preparation, and sequencing

Peripheral blood was collected from five hens per group at the end of the experimental trial conducted at the SARABiO Institute. Peripheral blood mononuclear cells (PBMCs) were isolated by density-gradient centrifugation using Lymphoprep (Serumwerk Bernburg AG, Bernburg, Germany), collected as cell pellets, and stored at −80°C until RNA extraction. Total RNA, including small RNAs, was extracted using the miRNeasy Mini Kit (QIAGEN, Hilden, Germany) according to the manufacturer’s instructions. RNA concentration was measured using a Qubit fluorometer (Thermo Fisher Scientific, Waltham, MA, USA), and RNA integrity and size distribution were evaluated using the RNA High Sensitivity assay on a TapeStation system (Agilent Technologies, Santa Clara, CA, USA). Based primarily on RNA integrity and electrophoretic profiles, four samples from each group were selected for RNA sequencing. Thus, RNA-seq analysis was performed using four biological replicates per group. Strand-specific total RNA libraries were prepared using the Illumina Stranded Total RNA Prep with Ribo-Zero Plus kit (Illumina, San Diego, CA, USA) according to the manufacturer’s instructions. Libraries were sequenced on an Illumina NovaSeq X Plus platform to generate 150-bp paired-end reads, yielding approximately 34–54 million paired-end reads per sample.

### Read mapping and differential expression analysis

Raw sequencing reads were processed using CLC Genomics Workbench version 26.0.4 (QIAGEN Digital Insights, Aarhus, Denmark). Adapter sequences and low-quality bases were removed prior to analysis. Filtered paired-end reads were mapped to the Gallus gallus reference genome (GRCg7b) with the corresponding gene annotation using the RNA-Seq Analysis workflow implemented in CLC Genomics Workbench. Gene-level expression values were exported for downstream statistical analyses. Subsequent analyses were performed in R version 4.5.1. Genes with TPM values greater than 1 in at least two samples were retained for analysis. Principal component analysis (PCA), correlation analysis, hierarchical clustering, volcano plots, MA plots, and heatmaps were generated to evaluate global transcriptional differences between groups. Differential expression analysis was performed using the limma package. Multiple-testing correction was performed using the Benjamini–Hochberg method. No genes remained statistically significant at a false discovery rate (FDR) < 0.05 after multiple-testing correction. Therefore, as an exploratory analysis, genes with a nominal p < 0.05 and an absolute log_2_ fold change > 1 were considered candidate differentially expressed genes. Gene Ontology (GO) enrichment analysis was conducted using the gprofiler2 R package, including Biological Process (GO:BP), Molecular Function (GO:MF), Cellular Component (GO:CC), KEGG, and Reactome databases. Functional categories with an adjusted *p* value < 0.05 were considered significantly enriched.

### Statistical analysis

Changes in egg production parameters over time were compared between the control and RG92 groups using repeated-measures two-way analysis of variance (ANOVA) to characterize differences associated with treatment group, age, and their interaction. Because hens were housed under floor-rearing conditions and individual eggs could not be assigned to specific hens, longitudinal laying-performance parameters were calculated at the flock level. Daily hen-day egg production was calculated from the total number of eggs produced divided by the number of hens in each group, and broken egg, LL-size egg, and acceptable egg rates were calculated from the corresponding daily egg counts. Mean ± SE values were calculated from these daily flock-level measurements within the indicated age periods. Because only one flock-level experimental unit was available for each treatment, repeated daily measurements were not considered independent biological replicates of the dietary treatment. Accordingly, statistical analyses of longitudinal laying-performance data were regarded as exploratory and were used to characterize differences in temporal patterns between the two treatment groups rather than to establish independently replicated treatment effects. When a nominal treatment × age interaction was detected, the laying period was divided into early and late phases, or early, middle, and late phases, as appropriate, and differences in daily flock-level values between groups within each phase were evaluated using Student’s t-test. These phase-specific comparisons were exploratory, and nominal *p* values were interpreted cautiously because multiple comparisons were performed. For egg quality measurements, eggs were randomly collected from each treatment group because individual laying hens could not be identified. Individual eggs were treated as analytical observations and as subsamples of the corresponding treatment group rather than as independent biological replicates of the dietary treatment. Eggshell color was evaluated using five eggs per group, whereas internal egg quality measurements, including Haugh unit and yolk index, were performed using ten eggs per group. Haugh unit and yolk index were compared between groups using Student’s t-test, and differences in eggshell color among the young hen, control, and RG92 groups were analyzed using the Steel-Dwass test. Fresh fecal samples were randomly collected from each treatment group under floor-rearing conditions and could not be linked to individual hens. Each collected fecal specimen was treated as an analytical observation and as a subsample of the corresponding treatment group rather than as an independent biological replicate. Fecal short-chain fatty acid and lactate concentrations were compared between groups using Student’s t-test. To evaluate age-associated changes in laying performance during the late laying period, linear regression analyses were performed using data from 66 to 119 weeks of age, and differences in regression slopes between groups were assessed using a test for parallelism. These regression analyses were also interpreted as exploratory because only one flock-level experimental unit was available for each treatment. For all non-transcriptomic analyses, nominal p values are reported for exploratory purposes and were not interpreted as confirmatory evidence of independently replicated treatment effects. A nominal p value < 0.05 was used to identify exploratory between-group differences. Statistical procedures for RNA-seq analyses are described in Section 2.7. All statistical analyses were performed using KyPlot version 6.0 (KyensLab Inc., Tokyo, Japan), except for RNA-seq analyses, which were performed using R.

## Results

### The RG92-supplemented flock showed maintained egg production performance during the late laying period

Egg production performance was monitored throughout the experimental period in the control flock (10 hens) and RG92-supplemented flock (10 hens), and production parameters were summarized at 3-week intervals as the mean ± SE. The RG92-supplemented flock showed an earlier onset of egg laying than the control flock; egg production was first observed at approximately 18 weeks of age in the RG92 group, whereas it began at approximately 20 weeks of age in the control group (Fig. 1A). Consequently, hen-day egg production was temporarily higher in the RG92 group during the onset of the laying period. After reaching peak laying performance, hen-day egg production remained comparable between groups during the early laying period. However, an age-associated decline became apparent after approximately 65 weeks of age, and the RG92-supplemented flock maintained a higher hen-day egg production rate than the control flock during the late laying period (Fig. 1A). The broken egg rate remained low in both groups during the early laying period but progressively increased with advancing age, particularly in the control group (Fig. 1B). In contrast, this age-associated increase was less pronounced in the RG92 group. Similarly, although the proportion of LL-size eggs (≥70 g) increased with age in both groups, the increase was substantially less pronounced in the RG92 group (Fig. 1C). Consequently, the acceptable egg rate, defined as the proportion of non-broken eggs excluding LL-size eggs, declined markedly in the control group but was maintained at a higher level in the RG92 group throughout the late laying period (Fig. 1D). These findings indicate that RG92 supplementation primarily attenuated age-associated deterioration of laying performance and egg quality rather than increasing peak laying performance.

**Fig. 1:**
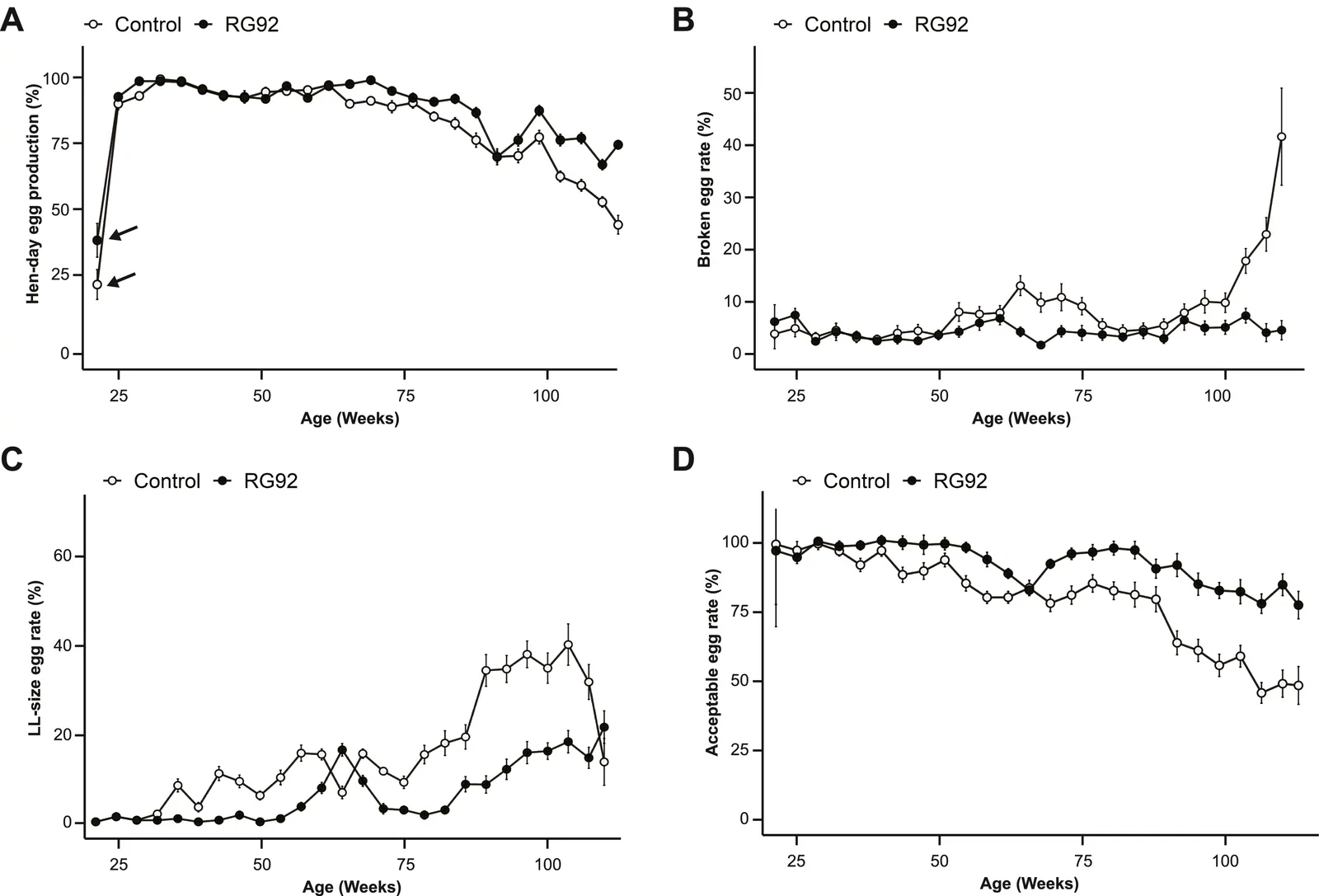
Longitudinal changes in egg production performance during the laying period. (A) Hen-day egg production, (B) broken egg rate, (C) LL-size egg rate (≥70 g), and (D) acceptable egg rate in the control (open circles) and RG92 (filled circles) groups from 18 to 119 weeks of age. Acceptable eggs were defined as non-broken eggs excluding LL-size eggs. Arrows in panel A indicate the earlier onset of egg laying in the RG92 group compared with the control group. Data are presented as the mean ± SE. Period-specific statistical comparisons are provided in Supplementary Data 1.

To quantitatively evaluate the rate of deterioration during the late laying period, regression analyses were performed from 66 to 119 weeks of age (Fig. 2). Hen-day egg production declined with age in both groups, whereas the rate of decline was less pronounced in the RG92 group (Fig. 2A). Conversely, the broken egg rate increased progressively in the control group but remained comparatively stable in the RG92 group (Fig. 2B). Similarly, the LL-size egg rate increased markedly with age in the control group but increased only gradually in hens receiving RG92 (Fig. 2C). Accordingly, the decline in the acceptable egg rate was less pronounced in the RG92 group (Fig. 2D). Period-specific statistical comparisons further supported these observations (Supplementary Data 1). Hen-day egg production was nominally higher in the RG92 group during multiple intervals between 66 and 119 weeks of age. The broken egg rate was nominally lower in the RG92 group during several late-laying intervals. Likewise, the LL-size egg rate remained nominally lower, whereas the acceptable egg rate remained nominally higher, in the RG92 group throughout most of the late laying period. These exploratory analyses were consistent with the observation that age-associated deterioration in laying performance and egg quality was less pronounced in the RG92-supplemented flock during the late laying period.

**Fig. 2:**
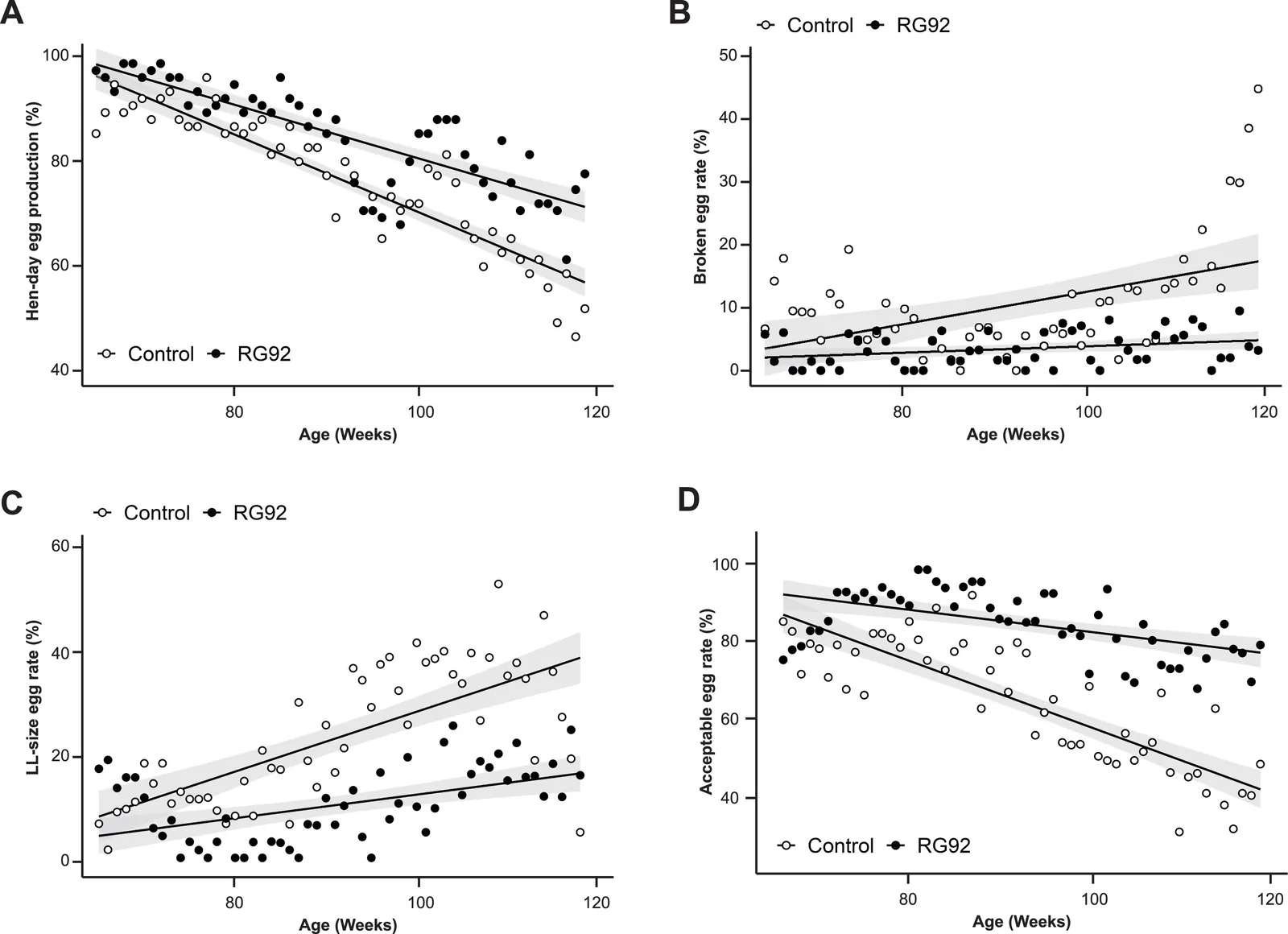
Linear regression analysis of egg production performance during the late laying period. Linear regression analyses of (A) hen-day egg production, (B) broken egg rate, (C) LL-size egg rate, and (D) acceptable egg rate from 66 to 119 weeks of age in the control (open circles) and RG92 (filled circles) groups. Shaded areas indicate the 95% confidence intervals of the regression lines. Differences in regression slopes between groups were evaluated using a test for parallelism.

### RG92-supplemented flock showed a less pronounced age-associated increase in egg weight and a more stable egg size distribution

Because egg weight was recorded individually from 80 weeks of age onward, changes in average egg weight were subsequently evaluated (Supplementary Fig. S1). Mean egg weight increased gradually with advancing age in both groups but remained consistently higher in the control group throughout the observation period. At 80 weeks of age, the average egg weight was 64.8 ± 0.4 g in the control group and 62.7 ± 0.4 g in the RG92 group. By the end of the experiment, the average egg weight reached 67.1 ± 0.7 g and 65.0 ± 0.8 g in the control and RG92 groups, respectively. To further characterize age-associated changes in egg size, eggs were classified into MS (52-57 g), M (58-63 g), L (64-69 g), and LL (≥70 g) size categories (Fig. 3A-D). In the control group, the proportion of M-size eggs gradually decreased with advancing age, whereas the proportion of LL-size eggs increased markedly during the late laying period. Consequently, L- and LL-size eggs accounted for the majority of eggs at the end of the experimental period. In contrast, the RG92-supplemented flock maintained a higher proportion of M- and L-size eggs, while the increase in LL-size eggs was attenuated compared with control hens. To visualize the overall shift in egg size distribution during the late laying period, the proportions of each egg size category were additionally summarized for the early (80-100 weeks) and late (101-119 weeks) phases (Supplementary Fig. S2). These observations indicate that the age-associated shift toward larger egg sizes was less pronounced in the RG92-supplemented flock during prolonged laying.

**Fig. 3:**
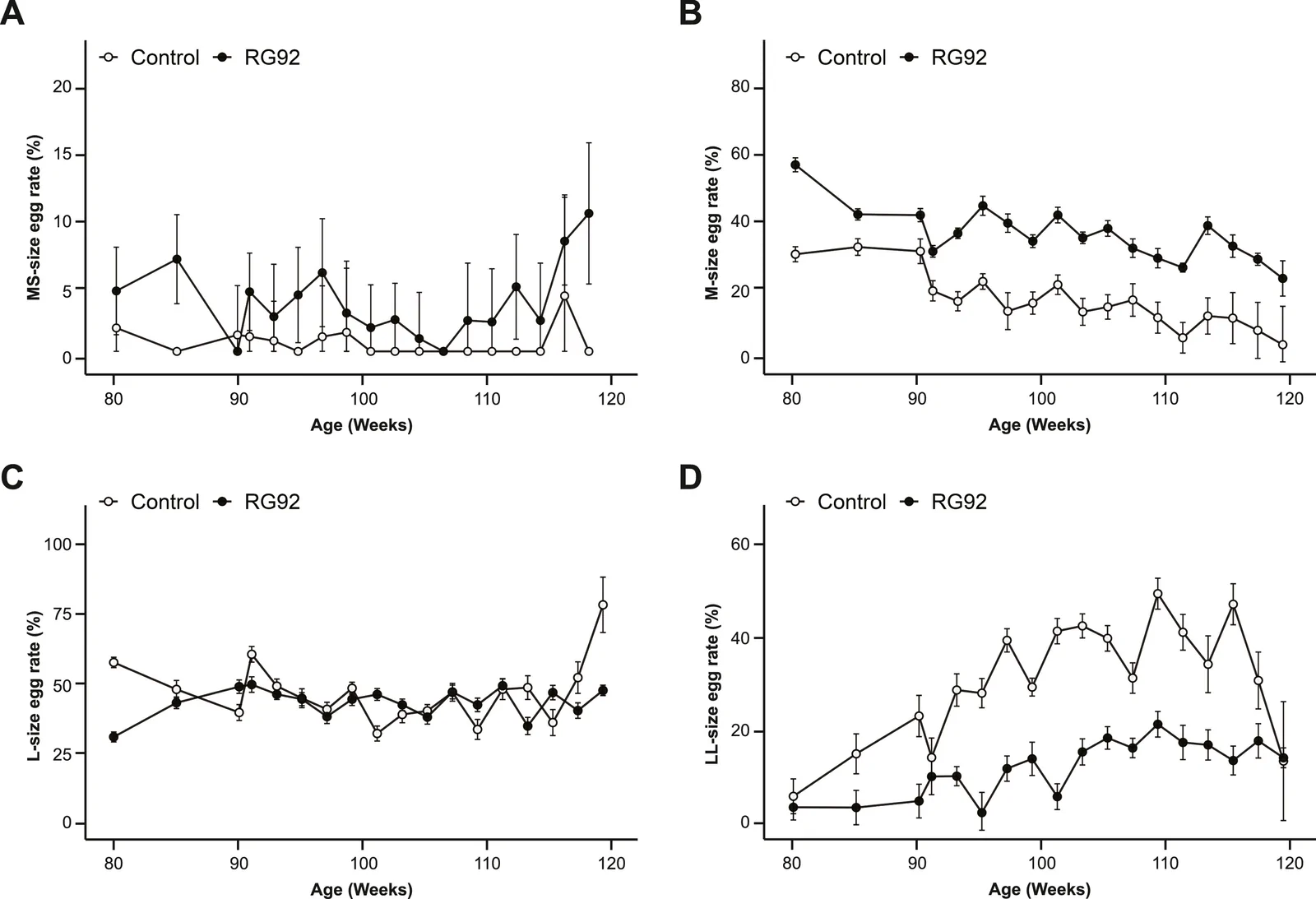
Age-dependent changes in egg size distribution during the late laying period. The proportions of (A) MS-size (52–57 g), (B) M-size (58–63 g), (C) L-size (64–69 g), and (D) LL-size (≥70 g) eggs from 80 to 119 weeks of age in the control (open circles) and RG92 (filled circles) groups. Data are presented as the mean ± SE.

### Commercial farm field evaluation showed a less pronounced age-associated decline in egg production in the RG92 group

To assess whether a similar pattern to that observed in the experimental trial was also evident under commercial farming conditions, a field trial was conducted at a commercial laying hen farm (Fig. 4). A total of 309 control hens and 289 RG92-supplemented hens were monitored from 48 to 78 weeks of age. Hen-day egg production gradually declined with advancing age in both groups; however, the decline appeared to accelerate in control hens during the latter part of the observation period, whereas the RG92 group maintained a relatively stable production rate (Fig. 4). Because the RG92 group showed slightly higher egg production at the beginning of the trial, the rate of age-associated decline was further evaluated by regression analysis. Linear regression analyses were performed separately for the early (48–63 weeks) and late (64–78 weeks) phases of the commercial trial. During the early phase, the regression slopes were comparable between groups, indicating comparable age-related changes in egg production. In contrast, during the late phase, the decline in hen-day egg production was nominally greater in control hens than in RG92-supplemented hens. These findings indicate that RG92 supplementation was associated with an attenuated age-associated decline in laying performance during the later phase of the commercial trial.

**Fig. 4:**
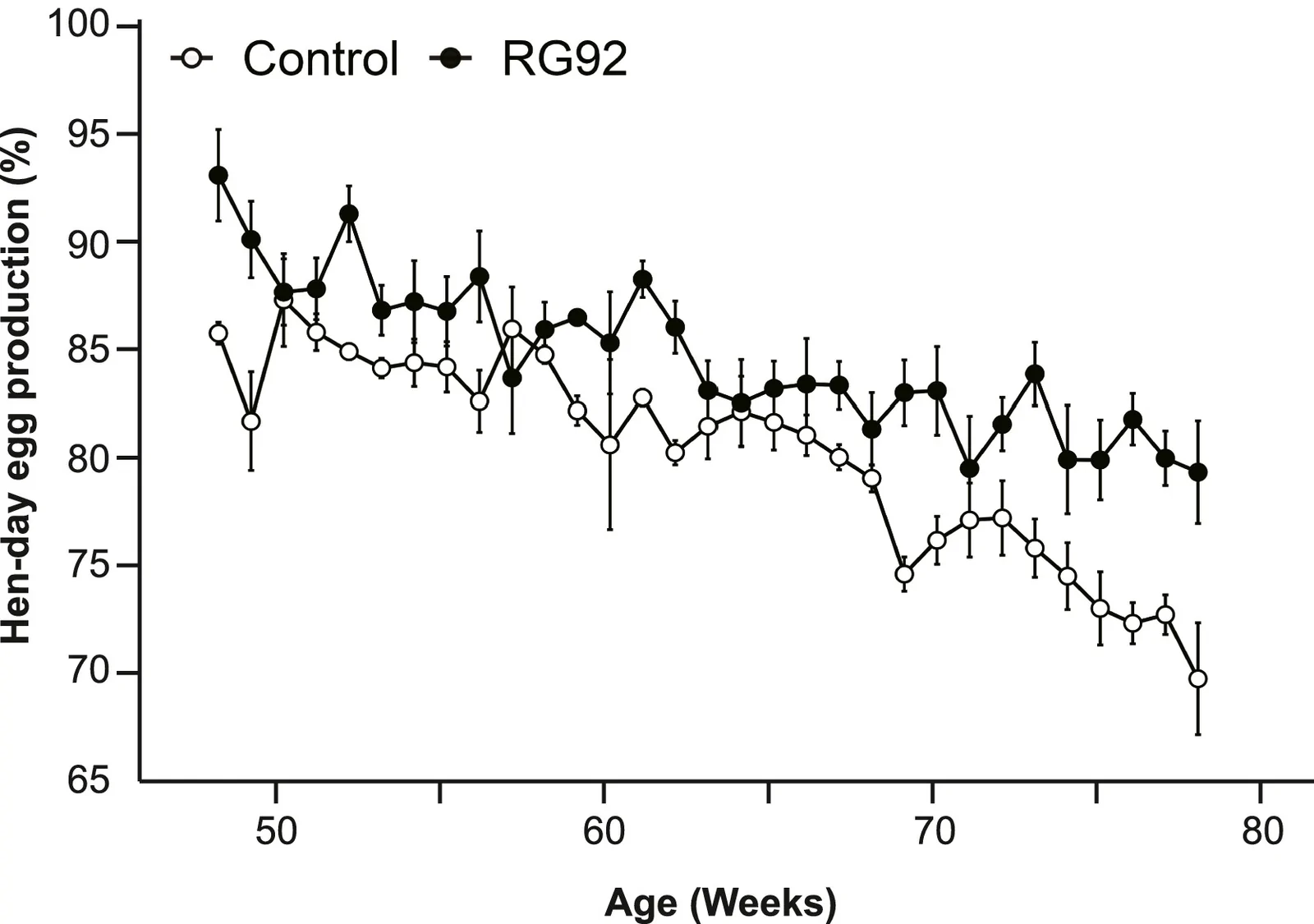
Hen-day egg production under commercial farming conditions. Hen-day egg production in the control (309 hens; open circles) and RG92 (289 hens; filled circles) groups from 48 to 78 weeks of age. Data are presented as the mean ± SE. Exploratory repeated-measures two-way ANOVA showed nominal effects of treatment, age, and the treatment × age interaction (all p < 0.001). Because differences between groups were already present during the early phase of the trial, age-associated changes in laying performance were further evaluated by regression slope analysis. Detailed statistical results are provided in Supplementary Data 1.

### RG92 attenuated age-associated deterioration of eggshell color and internal egg quality

Eggshell appearance and internal egg quality were evaluated at the end of the commercial farm field evaluation. Representative images showed that eggshell color was visibly darker in the RG92 group than in the control group (Fig. 5A). Quantitative evaluation using a shell color fan demonstrated that the eggshell color score was nominally higher in the RG92 group than in the control group and was comparable to that observed in young hens (Fig. 5B). These findings indicate that RG92 supplementation attenuated the age-associated loss of eggshell pigmentation. Representative images of eggs broken onto a flat surface showed that the thick albumen appeared more compact and elevated in the RG92 group than in the control group (Fig. 6A). The yolk index was higher in the RG92 group than in the control group (nominal *p* = 0.047; Fig. 6B). To further characterize yolk morphology, yolk diameter and yolk height were evaluated (Supplementary Fig. S3). Neither yolk diameter nor yolk height differed between groups, although the higher yolk index suggested better maintenance of yolk shape in RG92-supplemented hens.

**Fig. 5:**
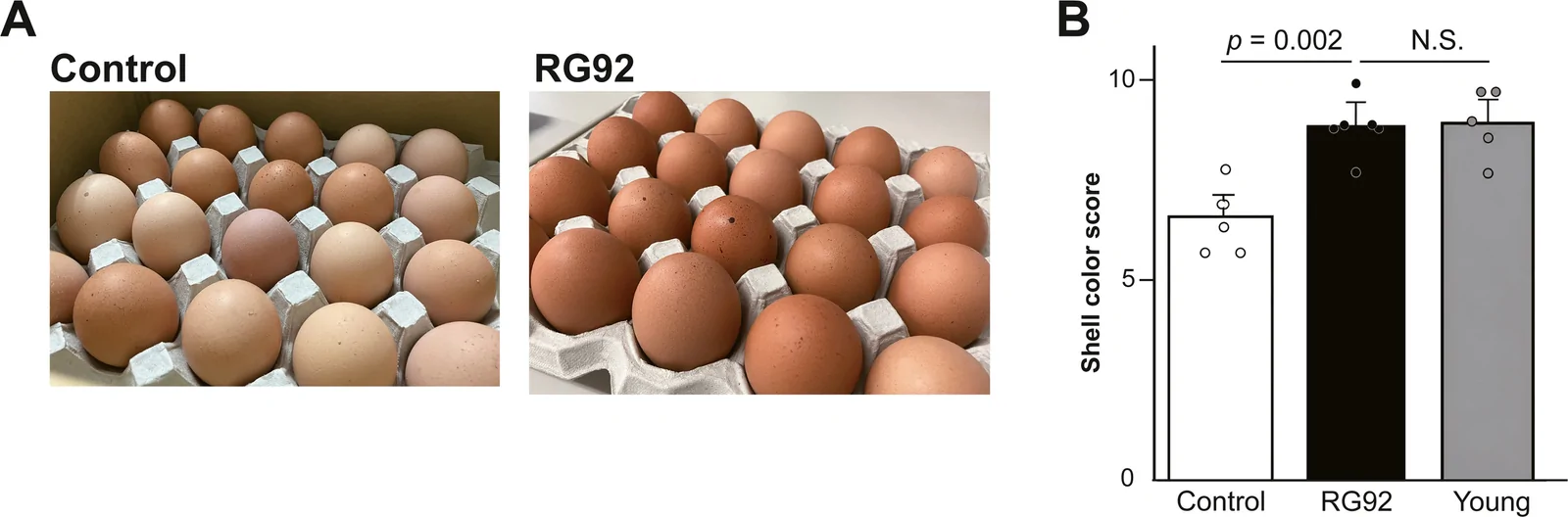
Dietary RG92 supplementation was associated with preserved eggshell pigmentation in aging laying hens. (A) Representative photographs of eggs collected from the control and RG92 groups at the end of the commercial farm field evaluation. (B) Eggshell color scores in the control, RG92, and young hen groups. Data are presented as the mean ± SE (n = 5 egg subsamples/group). Statistical differences among groups were evaluated using the Steel-Dwass test. N.S., not significant.

**Fig. 6:**
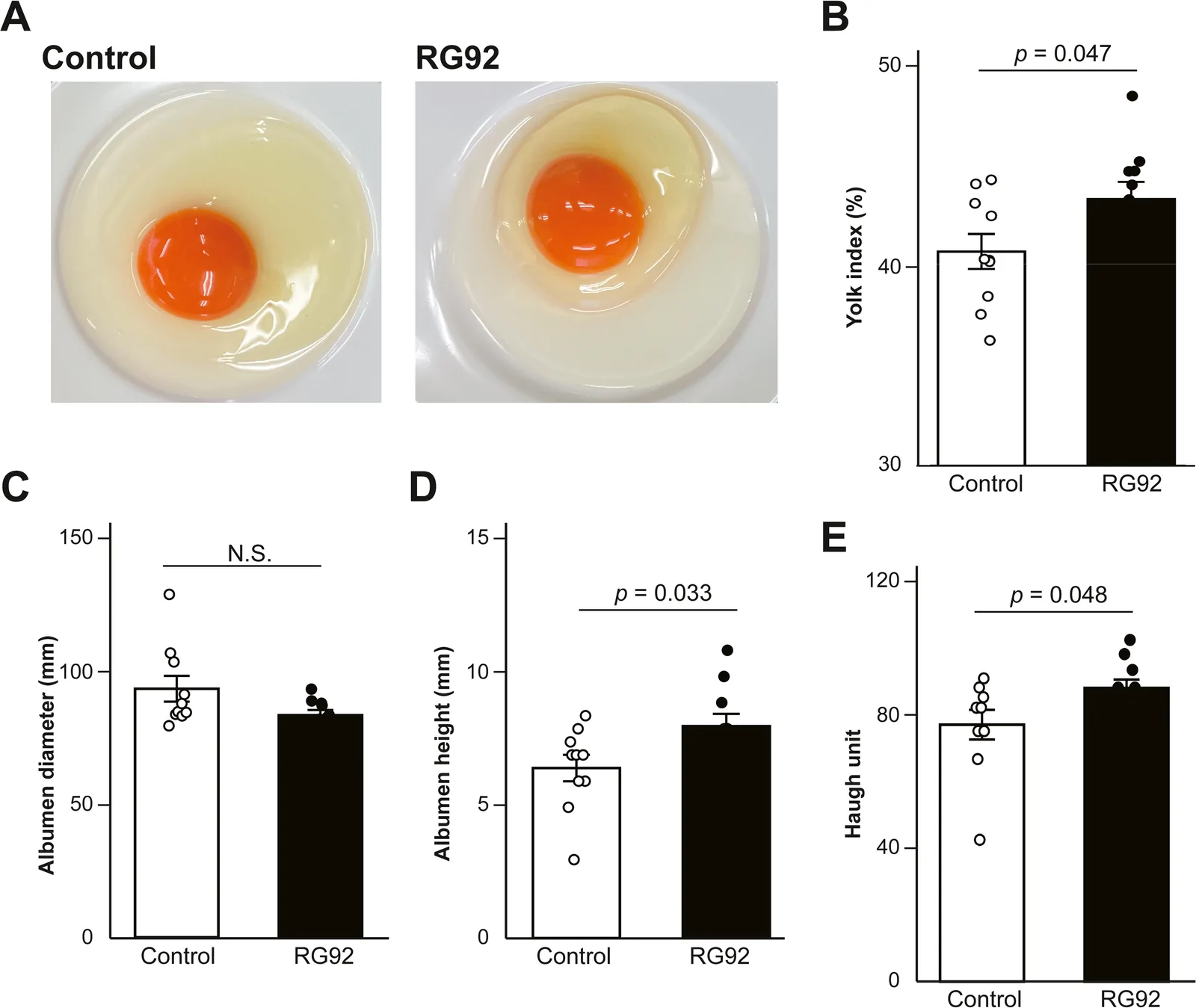
Internal egg quality at the end of the commercial farm field evaluation. (A) Representative photographs of eggs after cracking collected from the control and RG92 groups at 79 weeks of age. Quantitative comparisons of (B) yolk index, (C) albumen diameter, (D) albumen height, and (E) Haugh unit. Data are presented as the mean ± SE (n = 10 egg subsamples/group). Between-group comparisons were performed using Student’s t-test, and nominal p values are shown.

Albumen-related parameters were subsequently evaluated. Albumen diameter did not differ between groups (Fig. 6C), whereas albumen height was greater in the RG92 group (nominal *p* = 0.033; Fig. 6D). Consistent with the greater albumen height, the Haugh unit was also higher in the RG92 group (nominal *p* = 0.048; Fig. 6E). To further characterize the relationship between albumen morphology and quality parameters, albumen diameter and height were analyzed together (Supplementary Fig. S4). Albumen height tended to decrease as albumen diameter increased, suggesting that eggs with a more compact albumen structure tended to maintain greater albumen height. These findings suggest that RG92 supplementation was associated with better preservation of multiple age-related egg quality traits, including eggshell pigmentation, yolk shape, and albumen quality.

### RG92 supplementation was associated with higher fecal short-chain fatty acid concentrations

To characterize fecal metabolic changes associated with RG92 supplementation, fecal samples collected during the experimental period were analyzed for organic acid composition (Fig. 7). Acetic acid, propionic acid, and butyric acid concentrations were higher in the RG92 group than in the control group (nominal p < 0.05; Fig. 7A-C). Accordingly, total short-chain fatty acid (SCFA) concentration was also higher in the RG92 group (Fig. 7D). In contrast, lactate concentration tended to be lower in the RG92 group, whereas the total concentration of measured organic acids was comparable between groups (Fig. 7E-F). These findings suggest that RG92 supplementation was associated with a shift in the fecal organic acid profile rather than an overall increase in measured organic acid concentrations. To further characterize relationships among individual organic acids, correlation analyses were performed (Supplementary Fig. S5). Total SCFA concentration showed strong positive correlations with acetic acid, propionic acid, and butyric acid concentrations (Supplementary Fig. S5A-C). Positive correlations were also observed among the individual SCFAs (Supplementary Fig. S5D-F). Thus, the higher total SCFA concentration in the RG92 group was accompanied by increases in multiple SCFA components rather than a selective increase in a single metabolite. Because lactate can serve as a substrate for certain SCFA-producing bacteria, relationships between lactate and individual SCFAs were also examined (Supplementary Fig. S6). However, no significant correlations were observed between lactate and total SCFA, acetic acid, propionic acid, or butyric acid concentrations. These results do not support a simple relationship between fecal lactate depletion and increased SCFA concentrations. Further studies integrating microbial community analysis will be required to determine whether RG92 influences lactate-utilizing bacterial populations and their potential contribution to the observed fecal SCFA profile.

**Fig. 7:**
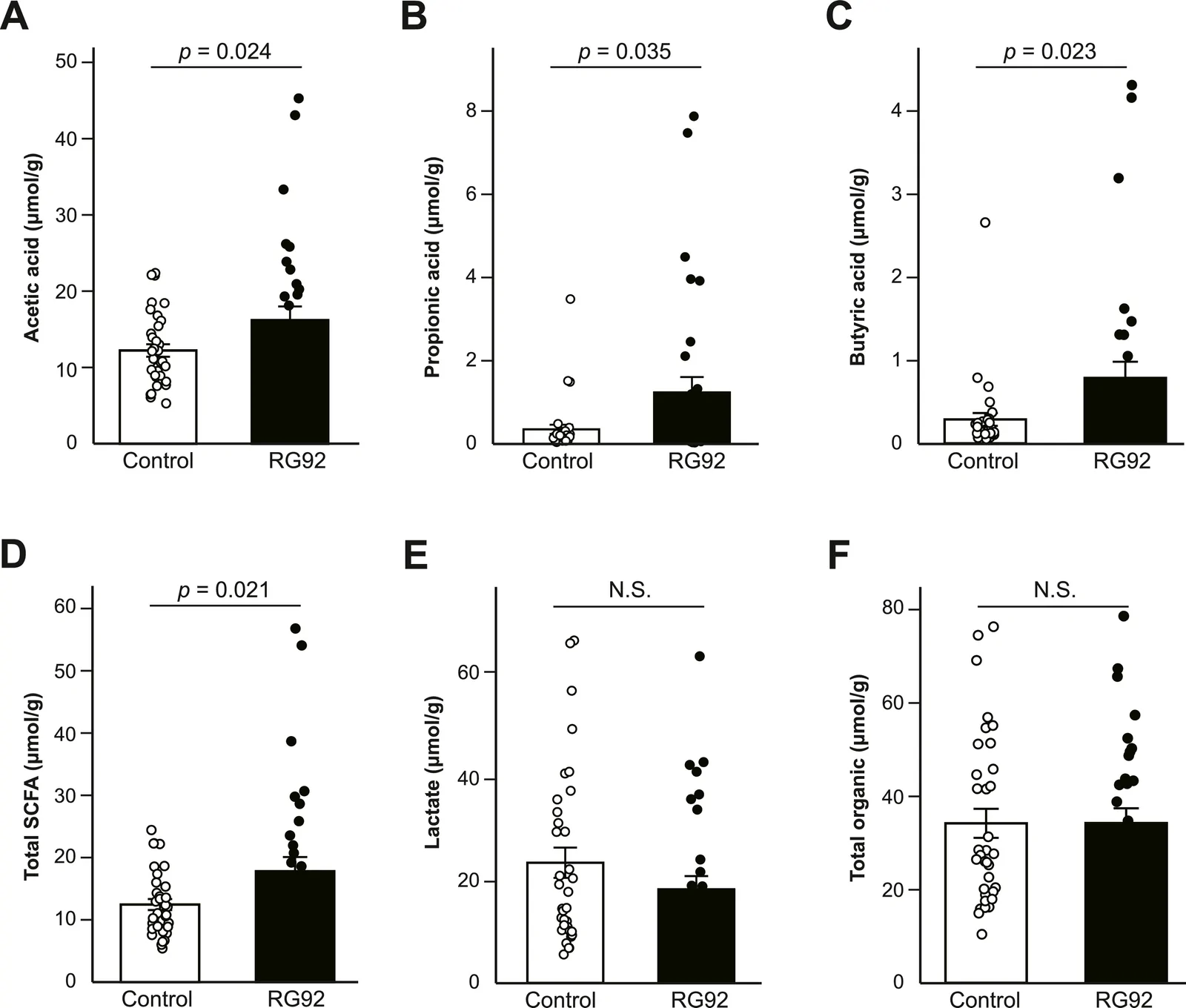
Fecal organic acid profiles in control and RG92-supplemented hens. Concentrations of (A) acetic acid, (B) propionic acid, (C) butyric acid, (D) total short-chain fatty acids (SCFAs), (E) lactate, and (F) total measured organic acids in fecal samples collected from the control and RG92 groups. Individual points represent fecal subsamples collected from the corresponding treatment groups, and bars represent the mean ± SE. Between-group comparisons were performed using Student’s t-test, and nominal *p* values are shown.

### RG92 supplementation was associated with PBMC transcriptomic differences

To investigate systemic molecular responses associated with RG92 supplementation, RNA sequencing was performed using peripheral blood mononuclear cells (PBMCs) collected from the control and RG92 groups. PBMCs were analyzed as a representative peripheral tissue to evaluate systemic responses to dietary intervention, including immune regulation and cellular homeostasis. First, overall differences in gene expression patterns among samples were evaluated using principal component analysis (PCA), which summarizes global transcriptomic variation and visualizes similarities and differences between samples. PCA showed a tendency toward separation between the control and RG92 groups, although greater inter-individual variability was observed within the RG92 group and some samples partially overlapped with the control group (Fig. 8A). Exploratory differential expression analysis identified multiple candidate genes showing expression differences between the RG92 and control groups based on nominal *p*-value and fold-change criteria (Fig. 8B). No individual genes remained statistically significant after correction for multiple testing (FDR < 0.05). Heatmap visualization of these candidate genes showed group-associated expression patterns while also reflecting inter-individual variability (Fig. 8C). Sample-to-sample correlation and Euclidean distance analyses further illustrated similarities and variability among individual transcriptomic profiles (Supplementary Fig. S7). Heatmaps of the top-ranked upregulated and downregulated candidate genes further illustrated expression patterns contributing to the observed group differences (Supplementary Figs. S8-S9). To explore the biological characteristics associated with these candidate transcriptional changes, Gene Ontology enrichment analysis was performed (Fig. 9). Enriched terms included immune response-regulating signaling pathway, immune response, regulation of immune response, and regulation of immune system process. In addition, terms related to cellular response to stimulus, ERBB signaling pathway, and epidermal growth factor receptor signaling pathway were enriched. These results indicate that the candidate transcriptional changes associated with RG92 supplementation were enriched for genes related to immune regulation and cellular response pathways.

**Fig. 8:**
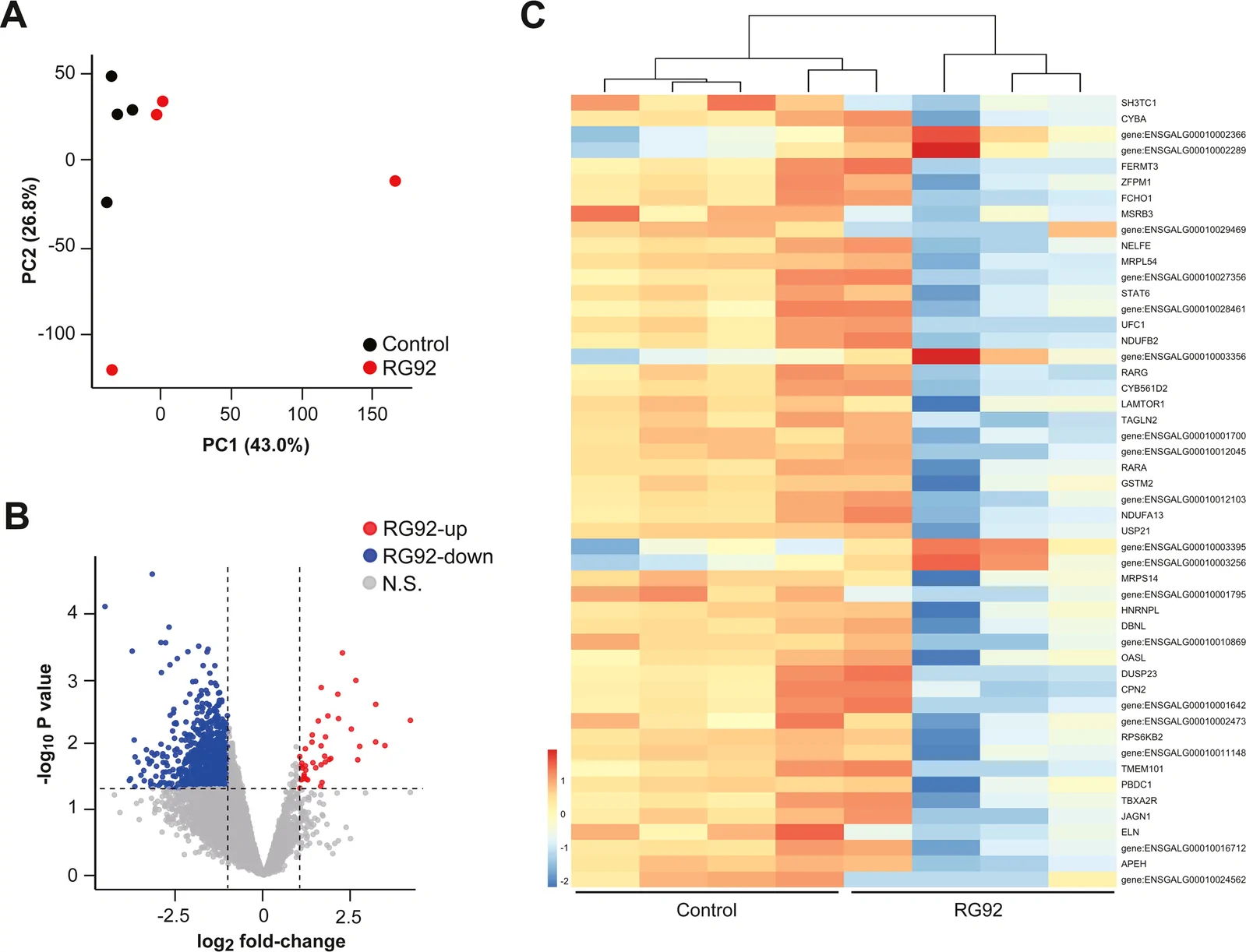
PBMC transcriptomic profiles associated with RG92 supplementation. (A) Principal component analysis (PCA) of PBMC RNA-seq data from the control and RG92 groups. (B) Volcano plot showing exploratory differential expression analysis between the control and RG92 groups. Red and blue dots indicate candidate genes showing higher and lower expression, respectively, in the RG92 group based on a nominal p < 0.05 and an absolute log₂ fold change > 1, whereas gray dots indicate genes not meeting these criteria. (C) Heatmap of candidate differentially expressed genes identified using these exploratory criteria. Gene expression levels are presented as row-wise Z-scores. Samples were hierarchically clustered using complete linkage. No individual genes remained statistically significant after multiple-testing correction at FDR < 0.05.

**Fig. 9:**
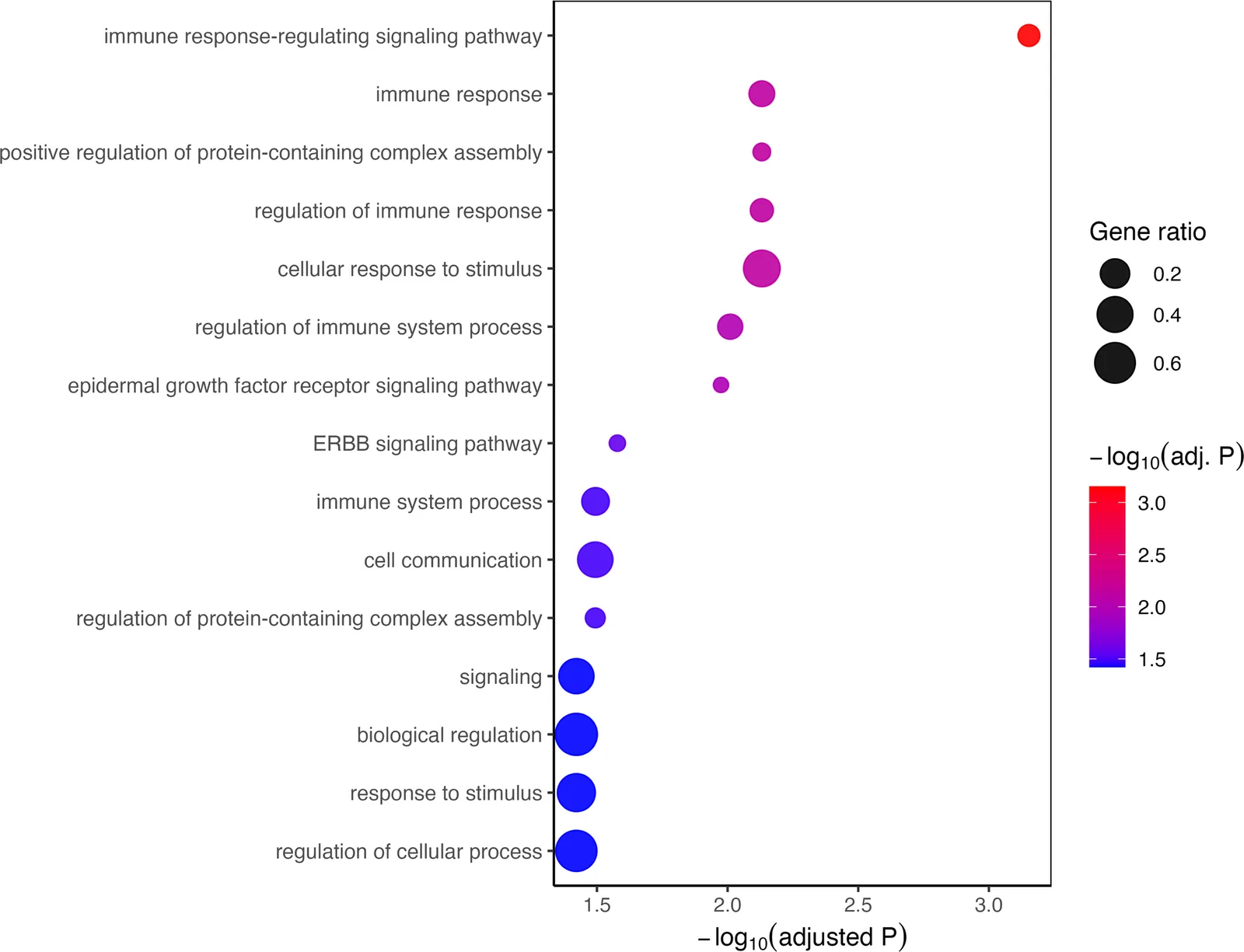
Gene Ontology enrichment analysis of candidate differentially expressed genes. Gene Ontology (GO) enrichment analysis based on candidate genes identified by the exploratory differential expression analysis between the control and RG92 groups. Bubble size represents the gene ratio, and color indicates the significance level (−log10 adjusted p value). Functional enrichment p values were adjusted for multiple testing.

### RG92 modulated immune- and oxidative stress-related gene expression

To further characterize the biological responses induced by RG92 supplementation, genes associated with immune regulation, oxidative stress, inflammasome signaling, innate immune signaling, histone deacetylase (HDAC)-related pathways, metabolic responses, and short-chain fatty acid transport were examined (Fig. 10A). Heatmap visualization of these functionally selected genes revealed coordinated changes in multiple biological pathways following RG92 supplementation. Among these genes, *STAT6* and *GLRX* were highlighted as representative genes because of their established roles in immune signaling and cellular redox homeostasis, respectively, and their relatively marked expression differences between the control and RG92 groups. *STAT6* showed lower expression in the RG92 group (nominal p = 0.019), whereas *GLRX* showed higher expression (nominal p = 0.035) (Fig. 10B and C). These nominal *p* values were not significant after correction for multiple testing and should therefore be interpreted as exploratory findings. Overall, these results suggest that RG92 supplementation was associated with alterations in immune- and oxidative stress-related transcriptional patterns in PBMCs.

**Fig. 10:**
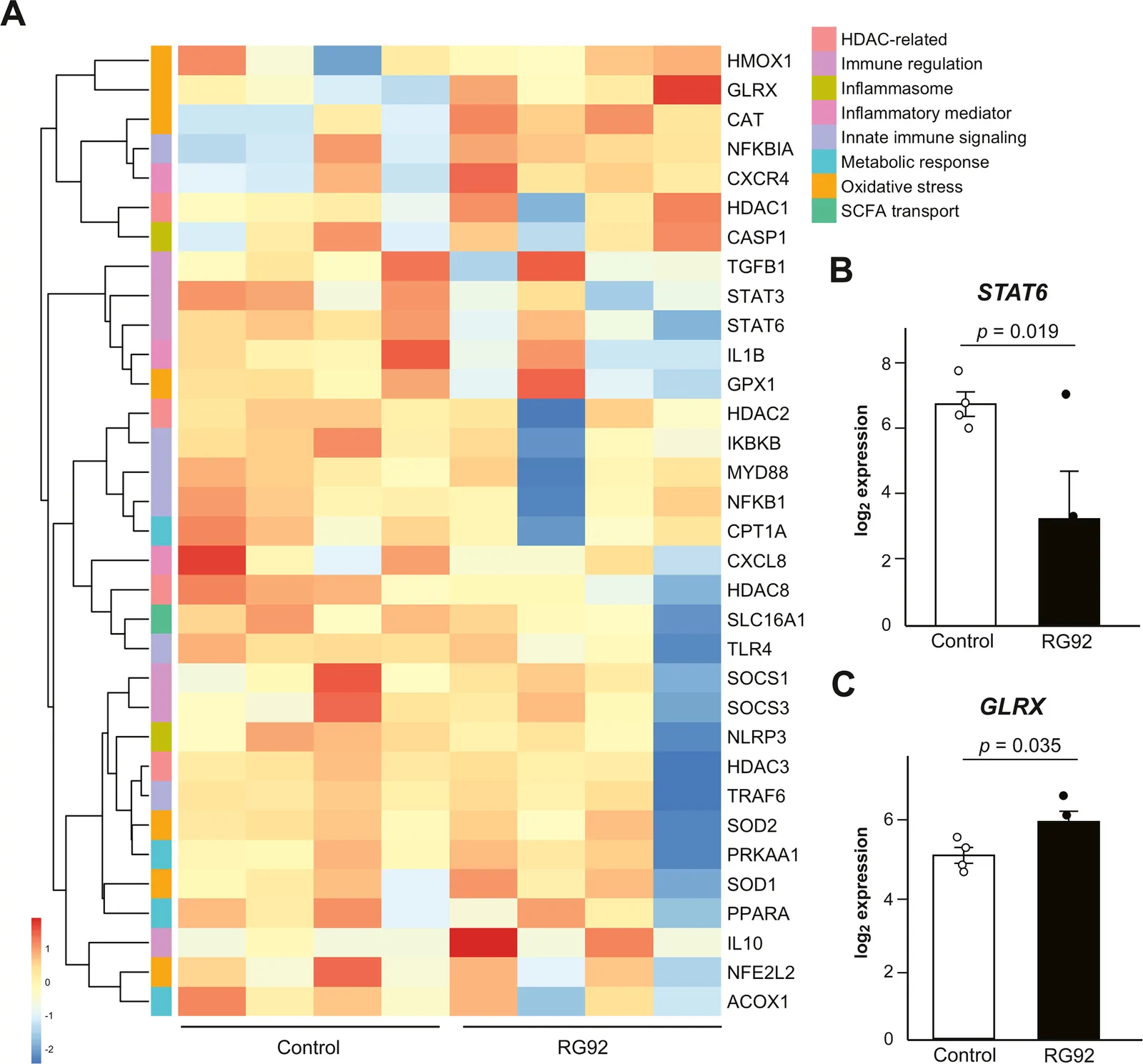
Expression patterns of selected immune- and oxidative stress-related genes associated with RG92 supplementation. (A) Heatmap of selected genes associated with immune regulation, inflammasome signaling, innate immune signaling, oxidative stress, histone deacetylase (HDAC)-related pathways, metabolic responses, and short-chain fatty acid transport. Gene expression levels are presented as row-wise Z-scores, and genes were hierarchically clustered according to their expression patterns. (B) Log_2_-transformed expression level of *STAT6*, a gene involved in immune signaling. (C) Log_2_-transformed expression level of *GLRX*, a gene involved in cellular redox homeostasis. Data are presented as the mean ± SE. Nominal *p* values were obtained from the limma differential expression analysis and were not statistically significant after correction for multiple testing.

## Discussion

The present study demonstrated that long-term dietary supplementation with RG92 improved both laying performance and egg quality in aging laying hens. Importantly, a similar pattern of a less pronounced age-associated decline in hen-day egg production was also observed under commercial farming conditions, indicating that the pattern observed in the experimental trial was also evident in a practical production setting. Compared with the control group, hens receiving RG92 maintained a higher hen-day egg production rate during the late laying period and exhibited fewer broken eggs and oversized (LL) eggs, resulting in a higher proportion of acceptable eggs. Furthermore, RG92 improved internal egg quality, as evidenced by increased albumen height, Haugh unit, and yolk index. Collectively, these findings indicate that RG92 may be associated with a less pronounced age-associated deterioration of productive performance and egg quality during prolonged laying.

The productive lifespan of commercial laying hens has gradually increased owing to advances in breeding and nutritional management (Arulnathan, et al., 2024). However, extending the laying cycle is inevitably accompanied by progressive deterioration in egg production and egg quality. As hens age, egg weight gradually increases, whereas eggshell characteristics, eggshell color, and internal egg quality, particularly albumen height and Haugh unit, progressively decline (Chang, et al., 2024; Kuttappan, et al., 2016; Rayan et al., 2010; Odabasi, et al., 2007; Silversides and Scott, 2001; Tumová and Gous, 2012). These age-associated changes increase the incidence of oversized and broken eggs, reduce the proportion of marketable eggs, and represent one of the major challenges facing the commercial egg industry. In the present study, the effect of RG92 on hen-day egg production became apparent during the late laying period, when age-associated deterioration in laying performance became evident in control hens. Rather than increasing peak egg production, RG92 supplementation attenuated the subsequent decline in laying persistency and reduced the deterioration of egg production parameters associated with aging. These findings suggest that the primary benefit of RG92 was not an increase in maximal productivity but a delay in age-related deterioration of laying performance and egg quality. One possible explanation for the stage-dependent pattern is that both groups maintained relatively high laying performance during the early laying period, leaving limited scope for a detectable difference between groups. As age-associated physiological deterioration became more pronounced during the later laying period, differences between the two treatment groups may have become more apparent, suggesting that RG92 may be more relevant to the maintenance of performance under age-related physiological challenge than to enhancement of peak production. From a practical perspective, these observations are particularly meaningful because the economic value of commercial egg production depends not only on the number of eggs produced but also on the proportion of eggs that meet quality standards. Oversized eggs are generally more susceptible to shell damage during production, transport, and grading, thereby increasing the likelihood of egg loss. Although the present study did not directly examine the causal relationship between oversized eggs and egg breakage, the simultaneous reduction in LL eggs and broken eggs, together with maintenance of a higher acceptable egg rate, suggests that RG92 may contribute to maintaining a higher proportion of eggs meeting the study-defined acceptable criteria during prolonged laying cycles.

Age-related deterioration of internal egg quality has been consistently reported in commercial laying hens. Progressive declines in albumen height and Haugh unit during the late laying period illustrate the marked deterioration of albumen quality associated with aging (Chang, Wang, Zhang, Qiu and Wu, 2024). A similar age-dependent decline in albumen height has also been observed regardless of genetic line (Silversides and Scott, 2001). Previous studies have demonstrated that functional feed additives can improve specific aspects of egg quality. For example, probiotic-fermented feed improved albumen height and Haugh unit (Guo, et al., 2021). In contrast, dietary supplementation with *Bacillus subtilis* increased the proportion of marketable eggs by reducing broken and soft-shelled eggs but did not significantly improve albumen height or Haugh unit (Zou, et al., 2021). Therefore, these findings suggest that different functional feed additives may preferentially improve different aspects of egg quality and production performance. In the present study, RG92 supplementation was associated with higher albumen height, Haugh unit, and yolk index in aged laying hens, suggesting partial preservation of internal egg quality during aging. In contrast, albumen diameter, yolk diameter, yolk height, and yolk color showed little or no response to supplementation, suggesting that RG92 selectively preserved egg quality traits that typically deteriorate with advancing age rather than broadly altering egg morphology. Notably, these differences were observed in aged laying hens, in which deterioration of egg quality is generally considered difficult to avoid. Together with the suppression of broken and oversized eggs, maintenance of a higher acceptable egg rate, and preservation of eggshell pigmentation, the present findings indicate that the effects associated with RG92 supplementation extend beyond sustaining egg production to preserving multiple egg quality traits during prolonged laying cycles.

Although the mechanisms underlying these beneficial effects remain to be fully elucidated, previous studies have demonstrated that RG92 possesses anti-inflammatory and metabolic regulatory properties. Oral administration of RG92 has been shown to improve insulin resistance and attenuate adipose tissue inflammation in experimental animals, while RG92 also directly suppresses inflammatory gene expression in cultured skin and articular cells (Kaseda, et al., 2020). These findings suggest that RG92 may modulate host physiological responses. In light of these previous findings, fecal short-chain fatty acids and PBMC transcriptomic profiles were evaluated to characterize physiological and molecular responses associated with RG92 supplementation. One of the notable findings of this study was an alteration in the fecal organic acid profile following RG92 supplementation. Fecal concentrations of acetate, propionate, and butyrate were higher in the RG92 group, whereas lactate tended to be lower, with no apparent difference in the total concentration of measured organic acids. These findings suggest that RG92 supplementation was associated with a shift in the fecal organic acid profile rather than an overall increase in measured organic acid concentrations. Short-chain fatty acids are major microbial metabolites that serve as energy substrates for intestinal epithelial cells and contribute to maintenance of intestinal physiology and host metabolism. Although the intestinal microbiota itself was not analyzed in the present study, the higher concentrations of multiple fecal SCFAs were consistent with an altered fecal metabolic profile associated with RG92 supplementation. Recent evidence indicates that gut microbiota-derived SCFAs not only regulate intestinal physiology but also exert systemic immunomodulatory effects after entering the circulation through activation of G protein-coupled receptors and inhibition of histone deacetylases (HDACs) (Ali, et al., 2022). Furthermore, SCFAs have been shown to modulate the function and transcriptional programs of peripheral immune cells, including peripheral blood mononuclear cells, through metabolic and epigenetic mechanisms (Wang, et al., 2026). In poultry, PBMC transcriptomic analyses have increasingly been used to characterize systemic immune responses, while recent studies further support communication between gut microbial metabolites and peripheral immune regulation (Chen, et al., 2025; Saint-Martin, et al., 2024).

Transcriptomic analysis further showed that RG92 supplementation was associated with alterations in PBMC gene expression. Principal component analysis showed a tendency toward separation between the RG92 and control groups, although inter-individual variability was evident, particularly within the RG92 group. Heatmap-based analyses of candidate genes also revealed group-associated expression patterns. Gene Ontology enrichment analysis of the candidate transcriptional changes identified biological processes related to immune regulation, immune responses, and responses to external stimuli, suggesting that RG92 supplementation was associated with transcriptional changes involving multiple aspects of immune regulation and cellular responses rather than a single molecular pathway. Among the functionally selected genes, *STAT6* and *GLRX* were highlighted as representative genes because of their established roles in immune signaling and cellular redox homeostasis, respectively. STAT6 is a key transcription factor activated by IL-4 and IL-13 signaling that regulates cytokine-mediated immune responses and immune homeostasis (Goenka and Kaplan, 2011), whereas GLRX is a glutathione-dependent oxidoreductase involved in the maintenance of cellular redox homeostasis and protection against oxidative stress (Ogata, et al., 2021). The *STAT6* mRNA showed lower expression, whereas *GLRX* showed higher expression, in the RG92 group based on nominal *p* values; however, neither remained statistically significant after correction for multiple testing. These observations should therefore be interpreted as exploratory, but they provide biologically relevant examples of the immune- and redox-related transcriptional patterns associated with RG92 supplementation. The simultaneous observation of higher fecal SCFA concentrations and altered PBMC transcriptomic profiles is consistent with emerging evidence suggesting interactions between the intestinal environment and systemic host physiology in laying hens. A comprehensive review proposed that intestinal microbiota and their metabolites, including short-chain fatty acids, may influence egg quality through the microbiota–gut–liver, microbiota–gut–brain, and microbiota–gut–reproductive tract axes, thereby affecting nutrient utilization, immune regulation, and reproductive physiology (Dai, et al., 2022). Furthermore, a study in late-phase laying hens demonstrated that rosemary extract improved Haugh unit together with enhanced intestinal barrier function, increased cecal butyrate production, altered gut microbial composition, and changes in immune- and albumen-related gene expression in the oviduct (Zhang, et al., 2023). Collectively, these previous findings support the possibility that nutritional modulation of the intestinal environment may be associated with systemic and reproductive responses relevant to egg quality.

Our observations are also consistent with previous studies reporting concurrent changes in host transcriptomic responses and intestinal microbial communities across different laying stages. Dynamic alterations in immune- and metabolism-related pathways have been reported alongside changes in the intestinal microbiota during the laying cycle, suggesting coordinated relationships between host transcriptional responses and microbial activity (Shi, et al., 2024). Although the intestinal microbiota itself was not characterized in the present study, the higher fecal concentrations of acetate, propionate, and butyrate together with the PBMC transcriptomic differences observed in RG92-supplemented hens suggest that RG92 may be associated with changes in both intestinal metabolic profiles and systemic host responses. Nevertheless, these findings should be interpreted with caution. Because neither gut microbial composition nor circulating metabolites were examined, causal relationships among microbial fermentation, SCFA production, PBMC transcriptional responses, and laying performance cannot be established from the present study. Future studies integrating microbiome profiling, metabolomics, and functional validation will be required to clarify the mechanisms linking RG92 supplementation with intestinal and systemic physiological responses and productive performance.

Several limitations of the present study should be considered. First, each treatment was represented by a single flock-level experimental unit in both the experimental and commercial studies; therefore, repeated longitudinal measurements do not constitute independent biological replication, and treatment-associated differences cannot be fully separated from possible flock- or rearing-section-specific effects. Second, eggs used for quality assessment and fecal specimens were collected as subsamples from the corresponding treatment groups and could not be linked to individual hens, precluding individual-level inference for these measurements. Third, the experimental and commercial studies differed in flock size, administration route, and supplementation level, and the commercial study should therefore be regarded as a supportive field evaluation rather than an independent replication of the experimental trial. These limitations indicate that the present findings should be confirmed in future studies using independently replicated flocks and designs that allow individual-level sampling where feasible.

## Conclusion

Dietary supplementation with RG92 was associated with a less pronounced age-associated decline in laying performance and was associated with preservation of multiple egg quality traits in aging laying hens throughout an extended laying period of up to 119 weeks. An attenuated age-associated decline in laying performance was also observed under commercial farming conditions, supporting the potential applicability of RG92 in prolonged laying cycles. RG92 supplementation was accompanied by alterations in fecal short-chain fatty acid profiles and PBMC transcriptomic responses related to immune regulation and cellular redox homeostasis, indicating concurrent intestinal metabolic and systemic transcriptional responses associated with RG92 supplementation**.** Although the underlying mechanisms remain to be fully elucidated, the present findings suggest that RG92 may help preserve productive performance and egg quality during extended laying periods and may represent a promising nutritional strategy for sustainable poultry production.

## Authorship contribution

Shigenori Nobata (S.N.) conceived the study together with the other investigators, coordinated the work with the poultry farm, prepared and cultured RG92, monitored body weight and egg production, evaluated egg quality, performed part of the statistical analyses, and contributed to the initial drafting and revision of the manuscript.

Sadahiro Iwabuchi (S.I.) was responsible for the overall statistical analyses, performed the RNA-seq analyses and interpretation, contributed to the initial drafting and revision of the manuscript, and provided financial support for the study.

Fumi Asao (F.A.) and Kenta Oka (K.O.) contributed to RG92 culture and preparation, coordinated the work with the poultry farm and feed company, collected samples, and critically reviewed and revised the manuscript for intellectual content.

Atsushi Tajima (A.T.) contributed to data analysis and interpretation, critically reviewed and revised the manuscript for important intellectual content, supervised the study, and provided financial support for the study.

Shigeru Hamada (S.H.) contributed to the original concept and study design, provided scientific advice throughout the study, and critically reviewed and revised the manuscript for important intellectual content.

All authors reviewed and explicitly approved the final version of the manuscript for publication. All authors agree to be personally accountable for all aspects of the work and to ensure that any questions related to the accuracy or integrity of the research are appropriately investigated and resolved.

## Funding

This work was supported by the Oita Prefectural Onsen Research/Study Committee, with funding awarded to S.H. The RNA sequencing analyses and publication costs were supported in part by the Kobayashi Foundation (Grant No. 425 to S.I.). Additional support was provided to A.T. through institutional research funds from Kanazawa University.

## Data availability

The raw FASTQ files generated by RNA sequencing in this study have been deposited in the DNA Data Bank of Japan (DDBJ) Sequence Read Archive (DRA) under the run accession numbers DRR1077408-DRR1077415.

## Ethics declaration

This study was conducted in accordance with the ARRIVE (Animal Research: Reporting of In Vivo Experiments) guidelines. This study was approved by the Animal Care and Use Committee of the SARABiO Institute.

## Scientific section

Metabolism and Nutrition

## Disclosures

Shigenori Nobata, Fumi Asao, Kenta Oka, and Shigeru Hamada are employees of SARABiO Onsen Probiotic Lab., Co., Ltd., which develops and commercializes RG92. Sadahiro Iwabuchi and Atsushi Tajima declare no competing financial interests or personal relationships that could have appeared to influence the work reported in this paper.

## Footnotes

Supplementary material associated with this article can be found, in the online version, at doi:10.1016/j.psj.2026.107972.
