# Supplementary material for: Dietary supplementation with RG92 improves laying performance and egg quality in laying hens
Source: Poult Sci. 2026 Oct 17;105(12):107972. doi: 10.1016/j.psj.2026.107972 (PMC13718793; doi:10.1016/j.psj.2026.107972)
Supplement: Supplementary file 1 [file mmc1.pdf]

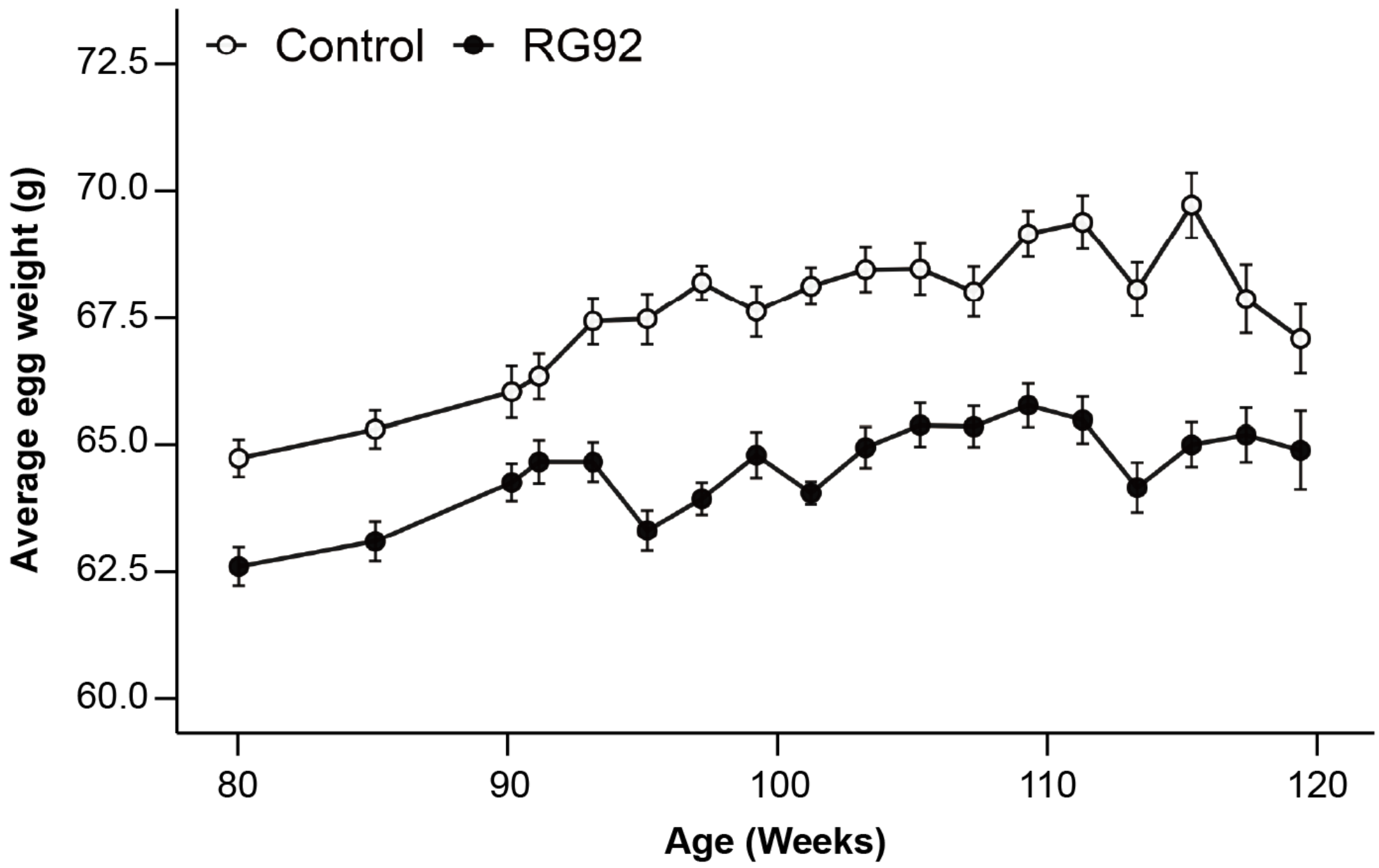

SUPPLEMENTARY FIGURE S1. Changes in average egg weight during the late laying period. Average egg weight in the control (open circles) and RG92 (filled circles) groups from 80 to 119 weeks of age. Data are presented as the mean ± SE.

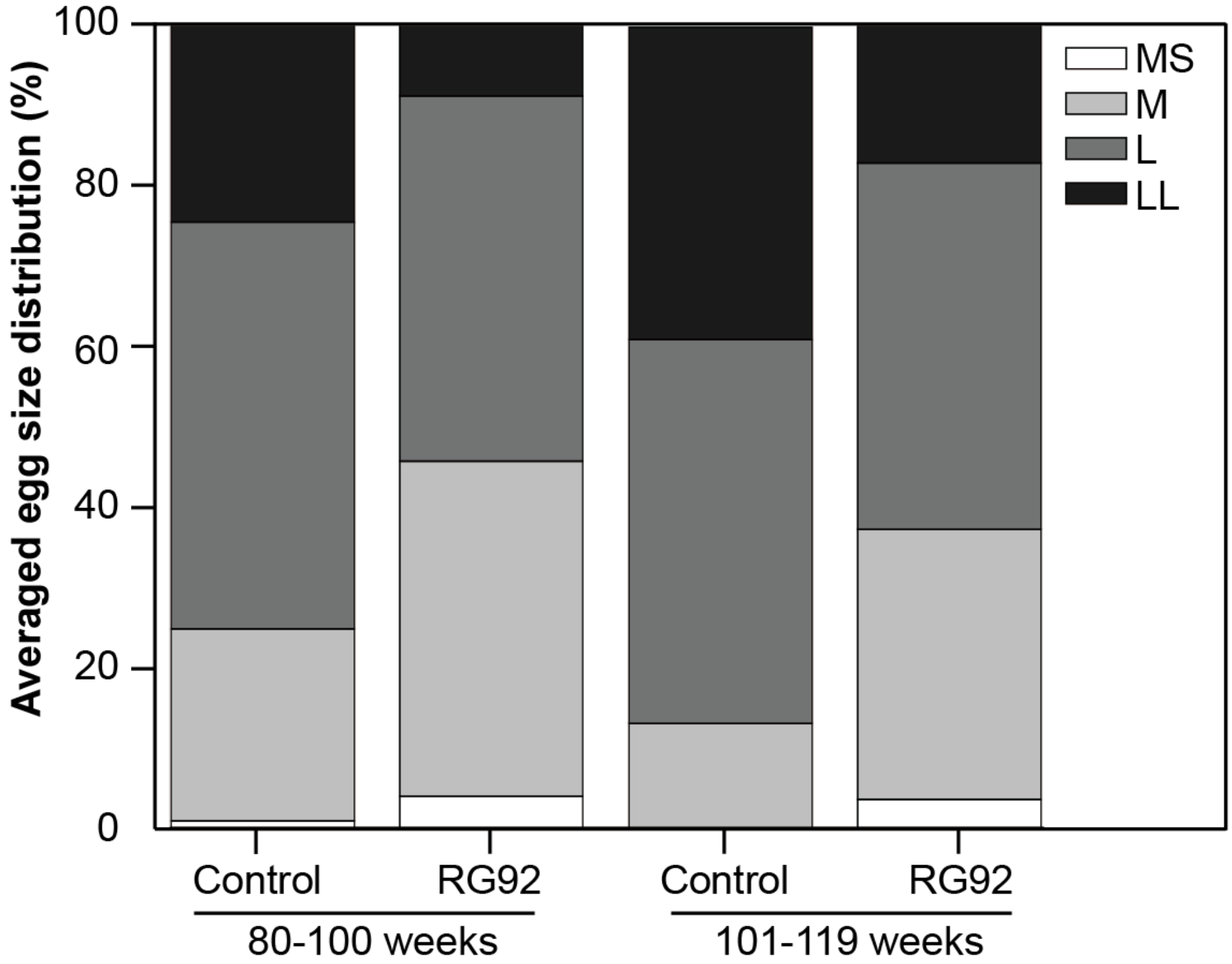

SUPPLEMENTARY FIGURE S2. Averaged egg size distribution during the late laying period. The average proportions of MS-size (52–57 g), M-size (58–63 g), L-size (64–70 g), and LL-size (≥70 g) eggs in the control and RG92 groups during 80–100 and 101–119 weeks of age. Each stacked bar represents the mean percentage distribution of egg size categories within the indicated period.

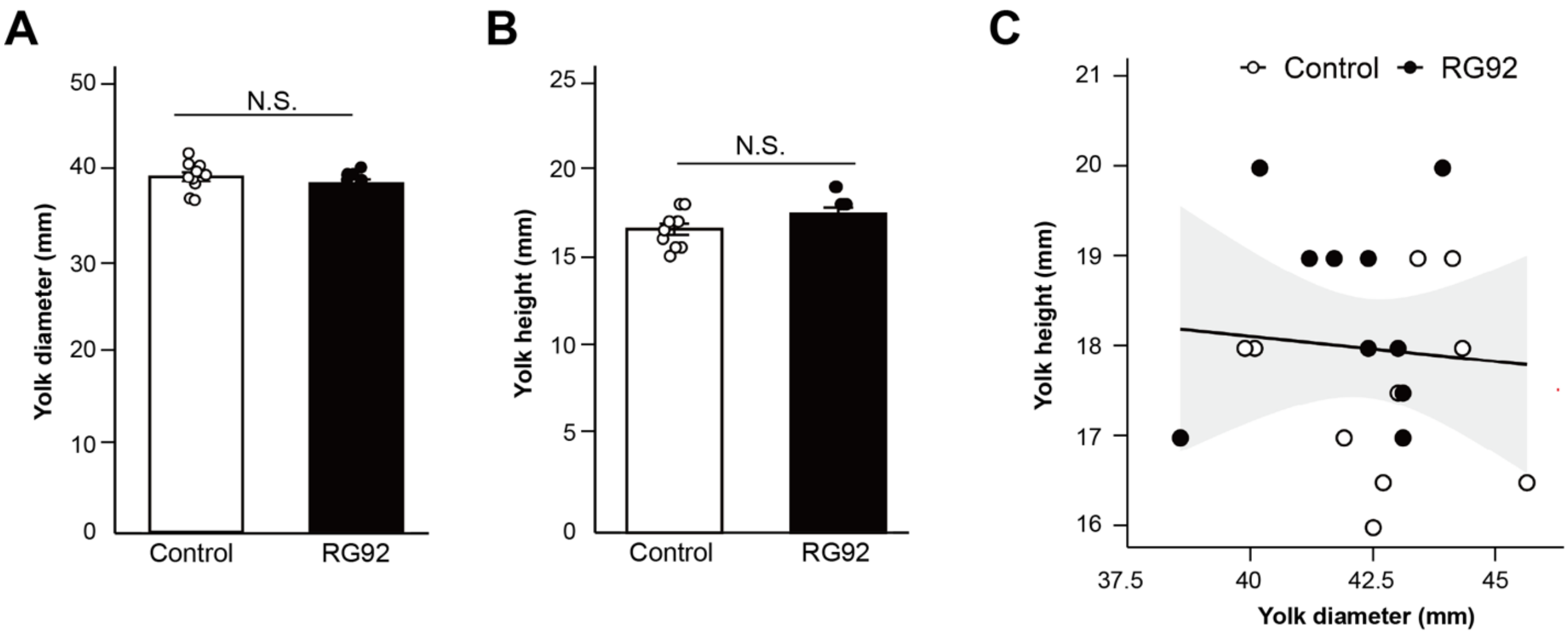

SUPPLEMENTARY FIGURE S3. Yolk morphology in the control and RG92 groups. (A) Comparison of yolk diameter between the control and RG92 groups. (B) Comparison of yolk height between the control and RG92 groups. (C) Correlation between yolk diameter and yolk height. The solid line represents the linear regression line, and the shaded area indicates the 95% confidence interval. N.S., not significant.

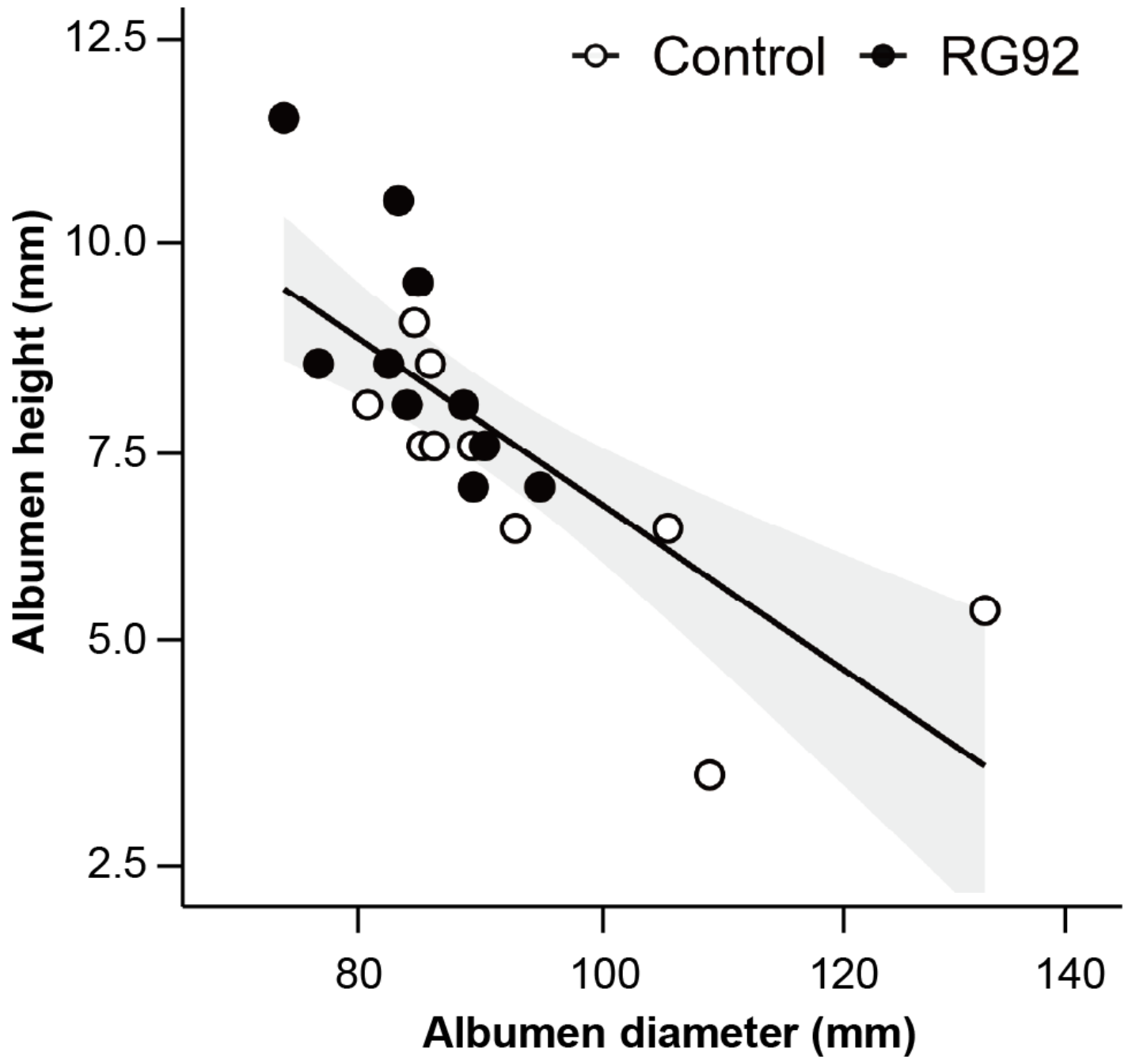

SUPPLEMENTARY FIGURE S4. Negative correlation between albumen diameter and albumen height. Correlation analysis of albumen diameter and albumen height in eggs from the control (open circles) and RG92 (filled circles) groups. Eggs with a larger albumen diameter tended to exhibit a lower albumen height. Each point represents an individual egg. The solid line indicates the linear regression line, and the shaded area represents the 95% confidence interval.

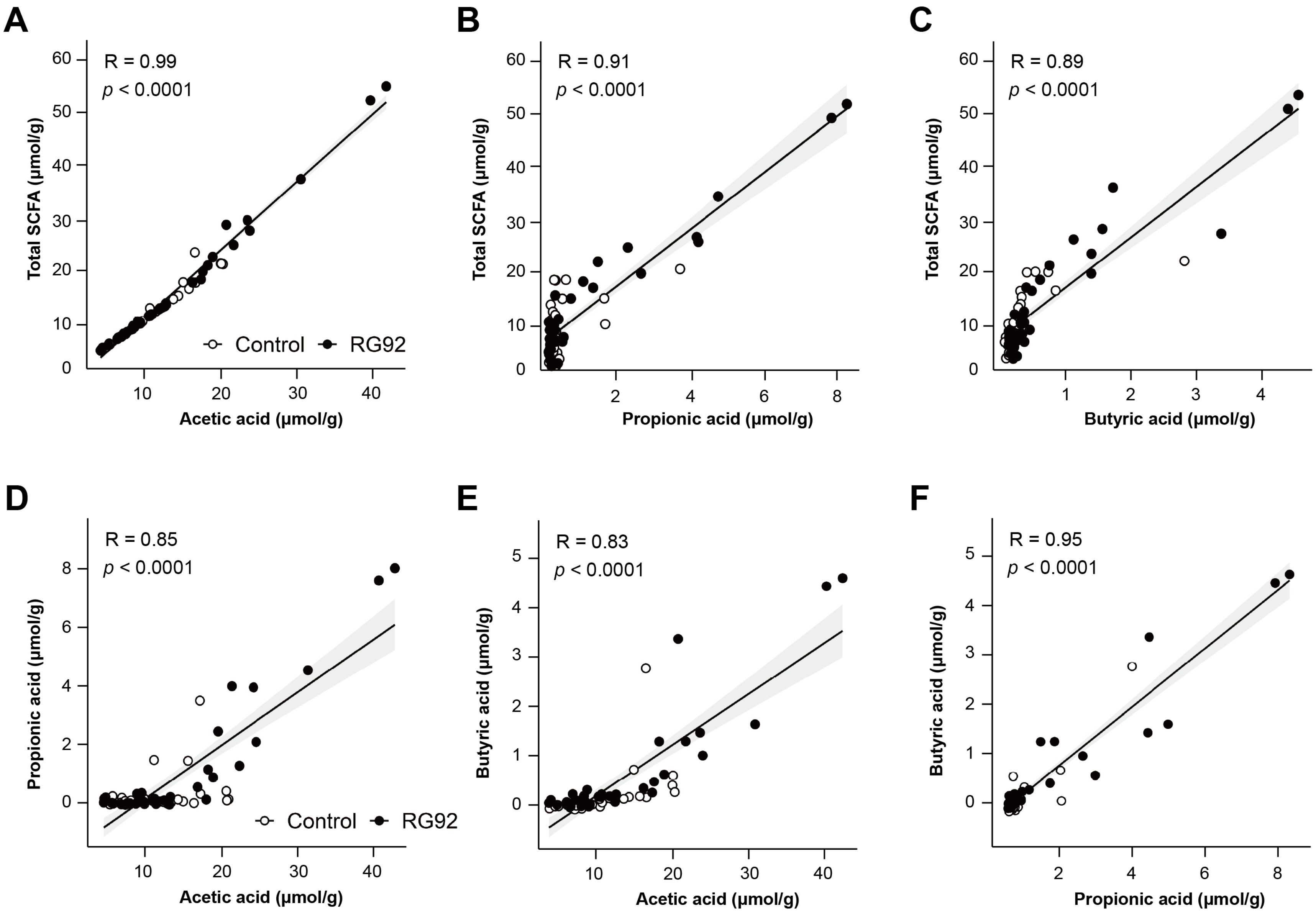
A
R = 0.99
p < 0.0001
Total SCFA (μmol/g)
Acetic acid (μmol/g)
Control
RG92
B
R = 0.91
p < 0.0001
Total SCFA (μmol/g)
Propionic acid (μmol/g)
C
R = 0.89
p < 0.0001
Total SCFA (μmol/g)
Butyric acid (μmol/g)
D
R = 0.85
p < 0.0001
Propionic acid (μmol/g)
Acetic acid (μmol/g)
Control
RG92
E
R = 0.83
p < 0.0001
Butyric acid (μmol/g)
Acetic acid (μmol/g)
F
R = 0.95
p < 0.0001
Butyric acid (μmol/g)
Propionic acid (μmol/g)

SUPPLEMENTARY FIGURE S5. Correlations among fecal short-chain fatty acid concentrations**.** Correlations between total short-chain fatty acid (SCFA) concentration and (A) acetic acid, (B) propionic acid, and (C) butyric acid concentrations, and correlations between (D) acetic acid and propionic acid, (E) acetic acid and butyric acid, and (F) propionic acid and butyric acid concentrations. Open and filled circles indicate samples from the control and RG92 groups, respectively. Solid lines represent linear regression fits, and shaded areas indicate the 95% confidence intervals. Pearson correlation coefficients (*R*) and corresponding *p* values are shown in each panel.

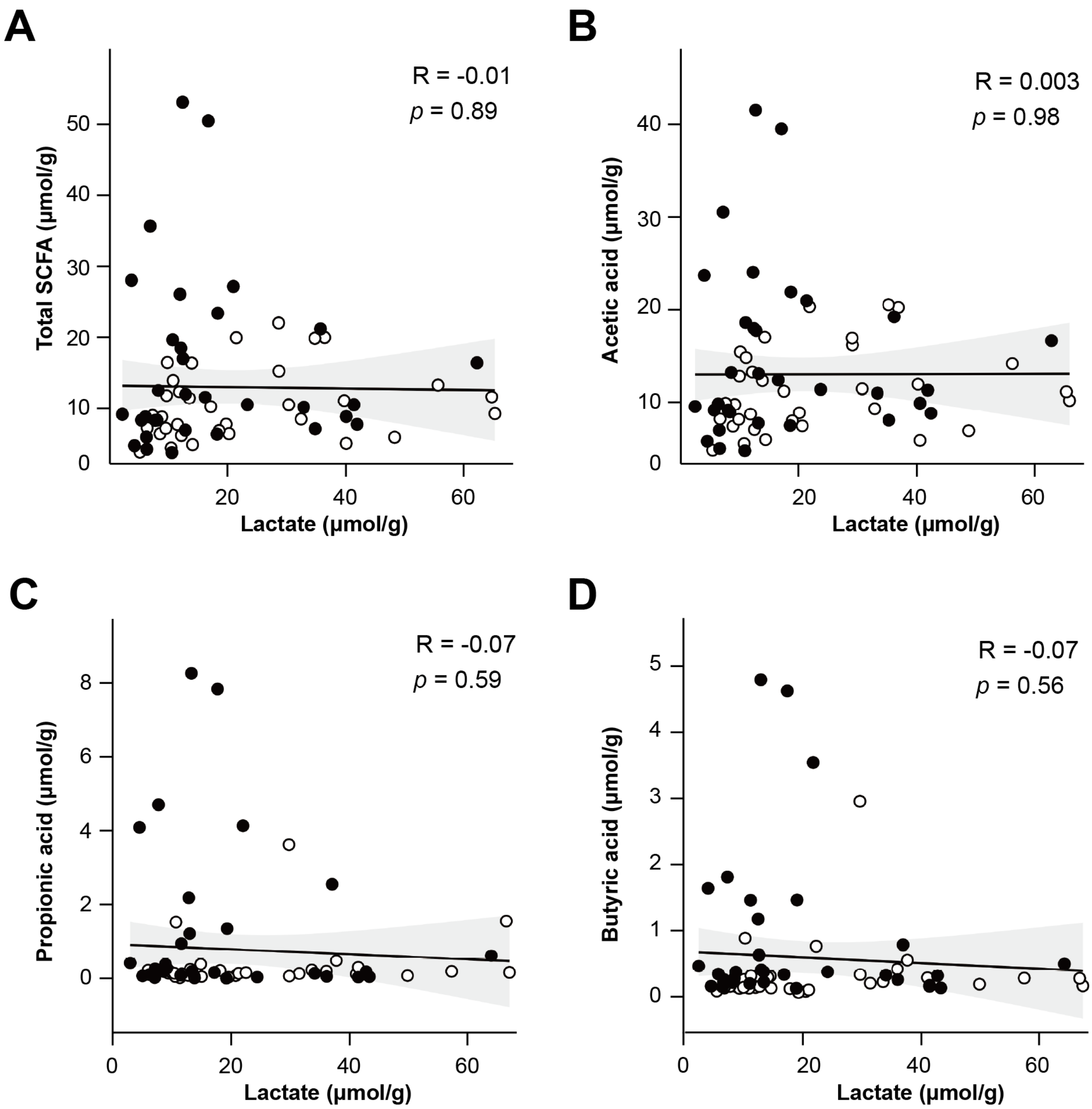

SUPPLEMENTARY FIGURE S6. Relationships between fecal lactate and short-chain fatty acid concentrations. Correlations between fecal lactate concentration and (A) total short-chain fatty acids (SCFAs), (B) acetic acid, (C) propionic acid, and (D) butyric acid concentrations. Open and filled circles indicate samples from the control and RG92 groups, respectively. Solid lines represent linear regression fits, and shaded areas indicate the 95% confidence intervals. Pearson correlation coefficients (*R*) and corresponding *p* values are shown in each panel.

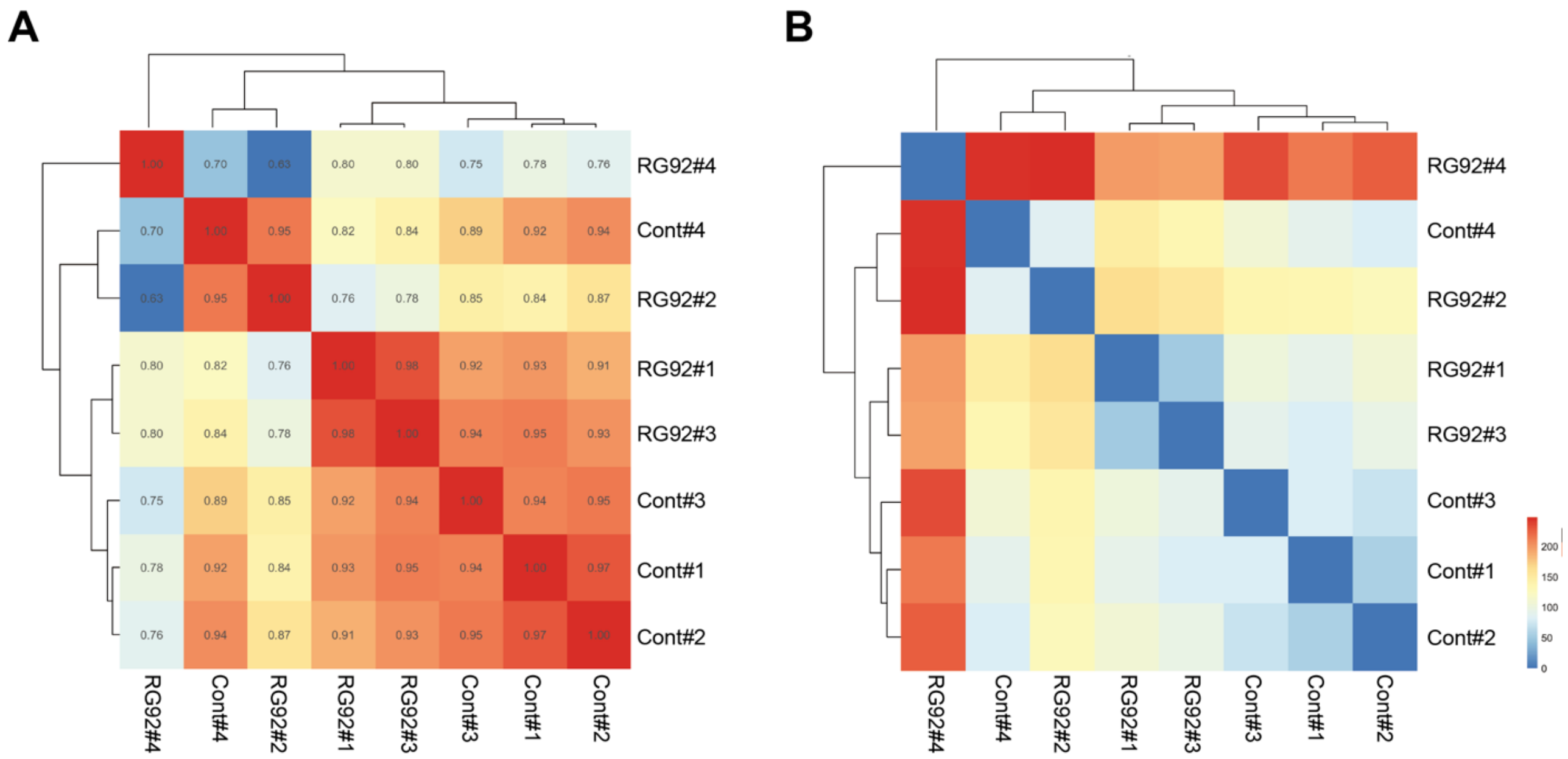

SUPPLEMENTARY FIGURE S7. Sample-to-sample correlation analysis of PBMC RNA-seq data. (A) Pearson correlation matrix showing pairwise correlations among individual RNA-seq samples from the control and RG92 groups. Numbers within each cell indicate Pearson's correlation coefficients. (B) Euclidean distance heatmap showing transcriptomic distances among samples. Hierarchical clustering was performed using complete linkage.

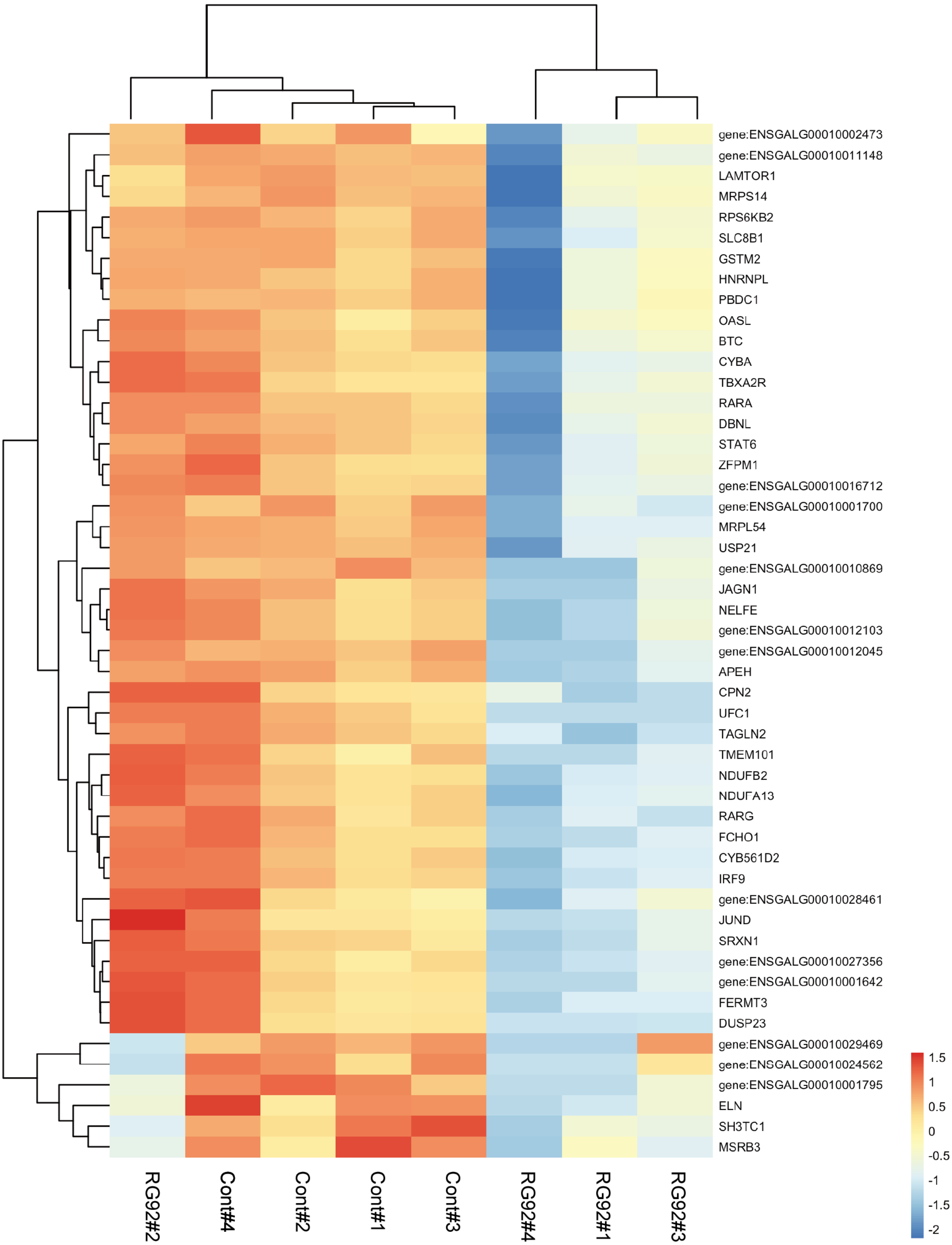
gene:ENSGALG00010002473
gene:ENSGALG00010011148
LAMTOR1
MRPS14
RPS6KB2
SLC8B1
GSTM2
HNRNPL
PBDC1
OASL
BTC
CYBA
TBXA2R
RARA
DBNL
STAT6
ZFPM1
gene:ENSGALG00010016712
gene:ENSGALG00010001700
MRPL54
USP21
gene:ENSGALG00010010869
JAGN1
NELFE
gene:ENSGALG00010012103
gene:ENSGALG00010012045
APEH
CPN2
UFC1
TAGLN2
TMEM101
NDUFB2
NDUFA13
RARG
FCHO1
CYB561D2
IRF9
gene:ENSGALG00010028461
JUND
SRXN1
gene:ENSGALG00010027356
gene:ENSGALG00010001642
FERMT3
DUSP23
gene:ENSGALG00010029469
gene:ENSGALG00010024562
gene:ENSGALG00010001795
ELN
SH3TC1
MSRB3
RG92#2
Cont#4
Cont#2
Cont#1
Cont#3
RG92#4
RG92#1
RG92#3
1.5
1
0.5
0
-0.5
-1
-1.5
-2

SUPPLEMENTARY FIGURE S8. Heatmap of the top 50 candidate genes with higher expression in the RG92 group. Heatmap showing the top 50 candidate genes with higher expression in the RG92 group than in the control group based on the exploratory differential expression analysis. Gene expression levels are presented as row Z-scores. Samples and genes were hierarchically clustered using complete linkage.

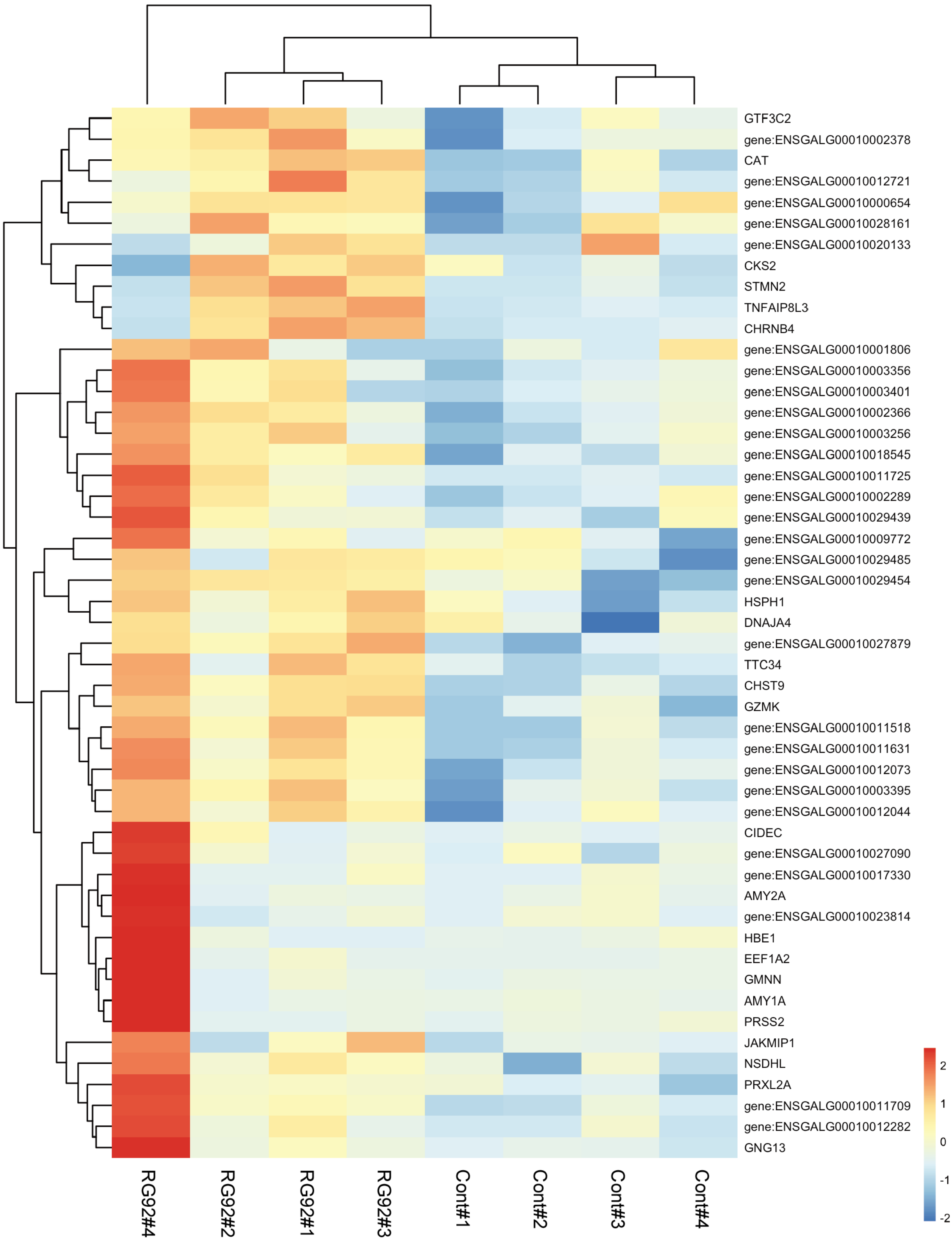
GTF3C2
gene:ENSGALG00010002378
CAT
gene:ENSGALG00010012721
gene:ENSGALG00010000654
gene:ENSGALG00010028161
gene:ENSGALG00010020133
CKS2
STMN2
TNFAIP8L3
CHRNB4
gene:ENSGALG00010001806
gene:ENSGALG00010003356
gene:ENSGALG00010003401
gene:ENSGALG00010002366
gene:ENSGALG00010003256
gene:ENSGALG00010018545
gene:ENSGALG00010011725
gene:ENSGALG00010002289
gene:ENSGALG00010029439
gene:ENSGALG00010009772
gene:ENSGALG00010029485
gene:ENSGALG00010029454
HSPH1
DNAJA4
gene:ENSGALG00010027879
TTC34
CHST9
GZMK
gene:ENSGALG00010011518
gene:ENSGALG00010011631
gene:ENSGALG00010012073
gene:ENSGALG00010003395
gene:ENSGALG00010012044
CIDEC
gene:ENSGALG00010027090
gene:ENSGALG00010017330
AMY2A
gene:ENSGALG00010023814
HBE1
EEF1A2
GMNN
AMY1A
PRSS2
JAKMIP1
NSDHL
PRXL2A
gene:ENSGALG00010011709
gene:ENSGALG00010012282
GNG13
RG92#4
RG92#2
RG92#1
RG92#3
Cont#1
Cont#2
Cont#3
Cont#4
2
1
0
-1
-2

SUPPLEMENTARY FIGURE S9. Heatmap of the top 50 candidate genes with lower expression in the RG92 group. Heatmap showing the top 50 candidate genes with lower expression in the RG92 group than in the control group based on the exploratory differential expression analysis. Gene expression levels are presented as row Z-scores. Samples and genes were hierarchically clustered using complete linkage.
